# v-Src-driven transformation is due to chromosome abnormalities but not Src-mediated growth signaling

**DOI:** 10.1038/s41598-018-19599-1

**Published:** 2018-01-18

**Authors:** Takuya Honda, Mariko Morii, Yuji Nakayama, Ko Suzuki, Noritaka Yamaguchi, Naoto Yamaguchi

**Affiliations:** 1 Laboratory of Molecular Cell Biology, Graduate School of Pharmaceutical Sciences, Chiba University, Chiba, 260-8675 Japan; 2 Department of Biochemistry and Molecular Biology, Kyoto Pharmaceutical University, Kyoto, 607-8414 Japan

## Abstract

v-Src is the first identified oncogene product and has a strong tyrosine kinase activity. Much of the literature indicates that v-Src expression induces anchorage-independent and infinite cell proliferation through continuous stimulation of growth signaling by v-Src activity. Although all of v-Src-expressing cells are supposed to form transformed colonies, low frequencies of v-Src-induced colony formation have been observed so far. Using cells that exhibit high expression efficiencies of inducible v-Src, we show that v-Src expression causes cell-cycle arrest through p21 up-regulation despite ERK activation. v-Src expression also induces chromosome abnormalities and unexpected suppression of v-Src expression, leading to p21 down-regulation and ERK inactivation. Importantly, among v-Src-suppressed cells, only a limited number of cells gain the ability to re-proliferate and form transformed colonies. Our findings provide the first evidence that v-Src-driven transformation is attributed to chromosome abnormalities, but not continuous stimulation of growth signaling, possibly through stochastic genetic alterations.

## Introduction

Src-family non-receptor-type tyrosine kinases play a key role in the regulation of diverse cellular functions, including cell proliferation, differentiation, adhesion, and motility^1–3^. c-Src, a member of the Src-family, is ubiquitously expressed in various types of tissues and cells. c-Src is overexpressed and abnormally activated in cancer cells, and the kinase activity of c-Src is correlated with progression of human cancers^4,5^. Although the activity of c-Src is tightly regulated in normal cells, deregulation of its activity often causes tumorigenesis. In particular, v-Src is the first identified oncogene product isolated from Rous sarcoma virus, and the kinase activity of v-Src is drastically increased compared with that of c-Src due to the presence of several point mutations and the lack of the C-terminal negative regulatory region^1,6–8^.

Colony formation assays have shown that expression of v-Src causes anchorage-independent and infinite cell proliferation. All of v-Src-expressing cells are supposed to form transformed colonies. However, abnormally low frequencies of the formation of v-Src-induced transformed colonies have been indicated in the literature^9–11^. These low frequencies of colony formation would be inconsistent with typical efficiencies of v-Src infection or transfection (approximately 5~80%).

In this study, to clarify a quantitative relationship between v-Src expression and colony formation, we utilized cell lines expressing tetracycline-inducible v-Src. Despite accomplishment of extremely high expression efficiencies of v-Src (nearly 100%), the frequencies of v-Src-induced colony formation were as low as those indicated in the literature. We revealed that inducible expression of v-Src up-regulates the cyclin-dependent kinase inhibitor p21, leading to cell cycle arrest, even though the ERK/MAPK pathway was simultaneously activated. We further showed that inducible expression of v-Src brings about chromosome abnormalities and approximately half of v-Src-expressing cells subsequently suppress v-Src expression. More importantly, a limited number of v-Src-suppressed cells can only start to re-proliferate vigorously and form transformed colonies. We thus provide evidence for a surprising role of v-Src in cell transformation.

## Results

### Cell cycle arrest by v-Src-induced tyrosine phosphorylation in HeLa S3 cells

In our recent study, we generated HeLa S3 and HCT116 cells, both of which express tetracycline-inducible wild-type v-Src (HeLa S3/TR/v-Src-wt and HCT116/TR/v-Src-wt), and showed that inducible expression of v-Src-wt forces the cells to become rounded and detached from a culture dish, which surprisingly leads to inhibition of cell proliferation in a manner dependent on the expression level of v-Src-wt^12^.

To examine the expression efficiency of v-Src-wt in HeLa S3/TR/v-Src-wt cells, we immunostained HeLa S3/TR/v-Src-wt cells with anti-Src (#327) antibody. Confocal microscopic and flow cytometric analyses showed the extremely high efficiency of v-Src-wt expression upon addition of doxycycline (Dox), a tetracycline derivative (Supplementary Fig. 1a,1b). Western blotting analysis confirmed Dox-dependent expression of v-Src-wt using anti-Src (N-16) antibody, which preferentially recognizes v-Src compared with c-Src (Supplementary Fig. 1c). Furthermore, using anti-Src (#327) antibody, which is capable of recognizing both c-Src and v-Src, we showed that the level of induced v-Src-wt expression was lower than that of endogenous c-Src. However, the level of protein tyrosine phosphorylation was dramatically increased by v-Src-wt expression due to the point mutations and the lack of the C-terminal negative regulatory region (Supplementary Fig. 1c). From 2 h after Dox addition, expression of v-Src-wt and tyrosine phosphorylation of cellular proteins were appreciably increased in a time-dependent manner (Supplementary Fig. 1d). These results suggest that HeLa S3/TR/v-Src-wt cells exhibit the extremely high efficiency of v-Src expression upon Dox treatment.

**Figure 1:**
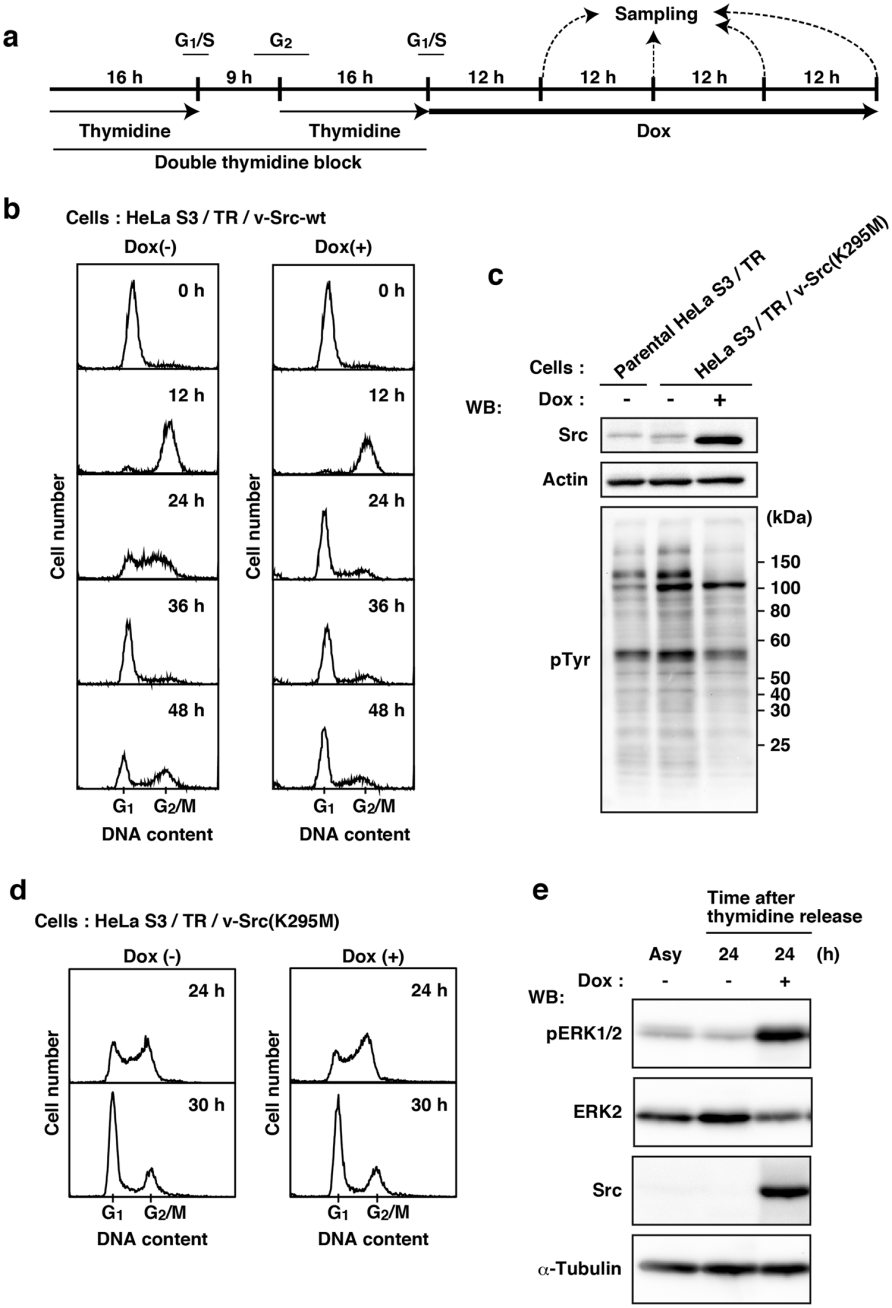
Cell cycle arrest in HeLa S3 cells by v-Src-induced tyrosine phosphorylation. (**a**) Schematic depiction of our synchronization method. Cells were synchronized by a double thymidine block and cultured for the indicated times in thymidine-free medium with or without 1 µg/ml Dox. (**b**) HeLa S3/TR/v-Src-wt cells were stained with propidium iodide (PI) for analyzing cell cycle progression by flow cytometry. The histogram [Dox(+), 0 h] is presented with the control histogram of untreated cells synchronized at the G_1_/S boundary [Dox(−), 0 h]. **(c)** HeLa S3 cells expressing inducible v-Src(K295M) [HeLa S3/TR/v-Src(K295M)] were cultured with or without 1 µg/ml Dox for 24 h, and whole cell lysates were analyzed by Western blotting (WB) using anti-Src (N-16), anti-actin, and anti-pTyr antibodies. (**d**) HeLa S3/TR/v-Src(K295M) cells were synchronized as depicted in Fig. 1a. Synchronized cells at the G_1_/S boundary were released for 24 h or 30 h with or without 1 µg/ml Dox and stained with PI for analyzing cell cycle progression by flow cytometry. (**e**) HeLa S3/TR/v-Src-wt cells were synchronized as depicted in Fig. 1a. Whole cell lysates were analyzed by Western blotting using anti-phospho-ERK1/2 (pERK1/2), anti-ERK2, anti-Src (N-16) and anti-α-tubulin antibodies. Asy stands for asynchronous HeLa S3/TR/v-Src-wt cells.

To examine the effect of v-Src on cell cycle progression, HeLa S3/TR/v-Src-wt cells were highly synchronized at the G_1_/S boundary by a double thymidine block (Fig. 1a). After release from the G_1_/S boundary, expression of v-Src-wt was induced by Dox treatment and DNA contents were analyzed by flow cytometry. v-Src-wt-expressing cells appeared to normally progress into G_2_/M phase 12 h after release from a double thymidine block (Fig. 1b). However, 24 h after the release, v-Src-wt expression inhibited cell cycle progression largely in G_1_ phase and slightly in G_2_ phase (Fig. 1b). To further examine whether the kinase activity of v-Src was indispensable to v-Src-mediated cell-cycle arrest, we generated a HeLa S3/TR cell line exhibiting inducible expression of v-Src(K295M), a kinase-dead mutant of v-Src [HeLa S3/TR/v-Src(K295M)] (Fig. 1c). Unlike v-Src-wt, expression of v-Src(K295M) did not affect cell cycle progression (Fig. 1d). These results suggest that v-Src expression causes cell cycle arrest in a manner dependent on the kinase activity.

It was reported that growth stimulation by v-Src expression requires activation of the mitogen-activated protein kinase (MAPK) pathway^13^. We were interested in examining whether ERK/MAPK signaling was activated during v-Src-mediated cell-cycle arrest. Intriguingly, phosphorylation of ERK at Thr-202 and Tyr-204 was greatly increased, indicative of activation of the ERK/MAPK pathway (Fig. 1e). Despite activation of the ERK/MAPK pathway through the Src-Ras-Raf-MEK-ERK signaling cascade^1,7,14^, these results suggest that v-Src expression causes cell cycle arrest against activated ERK-driven cell proliferation.

### Up-regulation of CDK inhibitor proteins by v-Src-induced tyrosine phosphorylation

**Figure 2:**
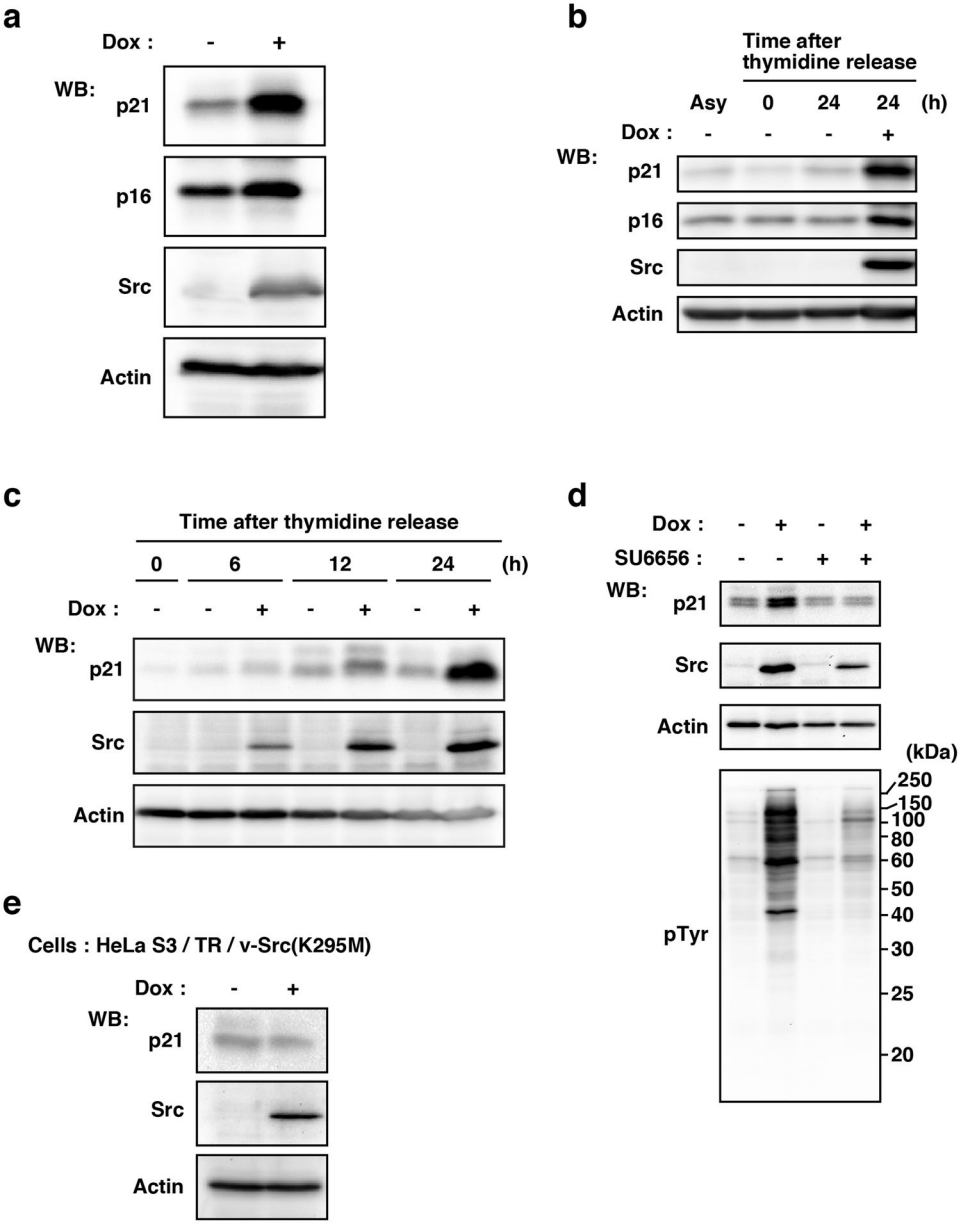
Up-regulation of CDK inhibitor proteins by v-Src-induced tyrosine phosphorylation. (**a**) HeLa S3/TR/v-Src-wt cells were cultured with or without 1 µg/ml Dox for 24 h, and whole cell lysates were analyzed by Western blotting (WB) using anti-p21, anti-p16, anti-Src (N-16), and anti-actin antibodies. (**b–d**) HeLa S3/TR/v-Src-wt cells were synchronized as depicted in Fig. 1a. (**b**) Whole cell lysates were analyzed by Western blotting using anti-p21, anti-p16, anti-Src (N-16), and anti-actin antibodies. Asy stands for asynchronous HeLa S3/TR/v-Src-wt cells. (**c**) Synchronized cells at the G_1_/S boundary were released for the indicated times with 1 µg/ml Dox, and whole cell lysates were analyzed by Western blotting using anti-p21, anti-Src (GD11), and anti-actin antibodies. (**d**) Synchronized cells at the G_1_/S boundary were cultured for 6 h with 1 μg/ml Dox in the presence or absence of 10 μM SU6656. Whole cell lysates were analyzed by Western blotting using anti-p21, anti-Src (N-16), and anti-actin antibodies. (**e**) HeLa S3/TR/v-Src(K295M) cells were cultured with or without 1 µg/ml Dox for 24 h. Whole cell lysates were analyzed by Western blotting using anti-p21, anti-Src (N-16), and anti-actin antibodies.

Cyclin-dependent kinase (CDK) inhibitor proteins, such as p21/Cip1 (p21) and p16/INK4a (p16), inhibit CDK activity, thereby blocking cell cycle progression^15^. We thus examined the levels of p21 and p16 and revealed that, 24 h after Dox addition, v-Src-wt expression up-regulated the levels of p21 and p16 (Fig. 2a,b). Furthermore, we kinetically examined the level of p21 after Dox addition, and showed that the level of p21 up-regulation was correlated with that of v-Src-wt expression (Fig. 2c). We then specifically inhibited the kinase activity of v-Src-wt using SU6656, a potent Src kinase inhibitor (see Supplementary Fig. S4d,S4e). Treatment with SU6656 was found to inhibit v-Src-wt-induced up-regulation of p21 (Fig. 2d). Moreover, expression of v-Src(K295M) did not increase the expression level of p21 (Fig. 2e). These results suggest that v-Src expression induces up-regulation of CDK inhibitor proteins in a manner dependent on v-Src kinase activity.

### Suppression of v-Src expression in NIH3T3 cells

**Figure 3:**
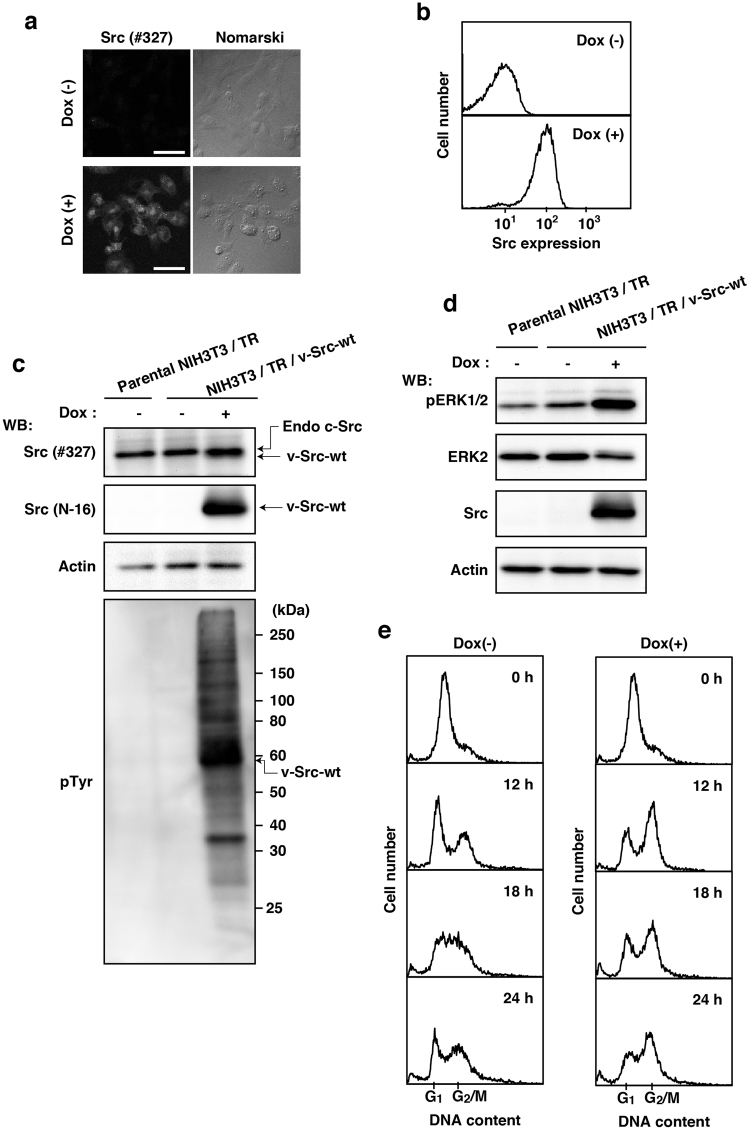
Delayed cell cycle progression in NIH3T3 cells by v-Src-induced tyrosine phosphorylation. (**a,b**) NIH3T3 cells expressing inducible v-Src (NIH3T3/TR/v-Src-wt) cultured with or without 1 µg/ml Dox for 24 h were fixed and stained with anti-Src (#327) antibody. (**a**) Confocal microscopic images and (**b**) flow cytometric histograms were shown. Scale bars, 50 µm. (**c,d**) Parental NIH3T3/TR and NIH3T3/TR/v-Src-wt cells were cultured with or without 1 µg/ml Dox for 24 h. (**c**) Whole cell lysates were analyzed by Western blotting (WB) using anti-Src (#327), anti-Src (N-16), anti-actin, and anti-pTyr antibodies. (**d**) Western blotting analysis was performed with anti-pERK1/2, anti-ERK2, anti-Src (N-16), and anti-actin antibodies. (**e**) NIH3T3/TR/v-Src-wt cells were synchronized as depicted in Fig. 1a. After release from synchronization, cells cultured with or without 1 µg/ml Dox for the indicated times were fixed and stained with PI for analyzing cell cycle progression. The histogram [Dox(+), 0 h] is presented with the control histogram of untreated cells synchronized at the G_1_/S boundary [Dox(−), 0 h].

Recently, we generated NIH3T3 cells expressing inducible v-Src-wt (NIH3T3/TR/v-Src-wt) and showed that, like HeLa S3 and HCT116 cells, v-Src-wt expression forces NIH3T3 cells to inhibit cell proliferation in a manner dependent on the expression level of v-Src-wt^12^. Thus, we analyzed cell cycle progression of NIH3T3/TR/v-Src-wt cells. NIH3T3/TR/v-Src-wt cells also showed the extremely high efficiency of v-Src-wt expression upon Dox treatment (Fig. 3a–c; see Supplementary Fig. 1). Next, we analyzed ERK activation in NIH3T3/TR/v-Src-wt cells and showed that v-Src-wt expression activated ERK upon Dox treatment (Fig. 3d). Moreover, we examined the effect of v-Src expression on cell cycle progression of NIH3T3 cells using a double thymidine block as depicted in Fig. 1a. In the absence of Dox, NIH3T3/TR/v-Src-wt cells were able to normally enter G_1_ phase 12 h after release from the G_1_/S boundary. However, inducible expression of v-Src delayed the transition from G_2_/M to G_1_ phase (Fig. 3e) and inhibited entry into M phase (Supplementary Fig. S6). These results suggest that v-Src expression inhibits cell cycle progression not only in HeLa S3 cells but also in NIH3T3 cells.

**Figure 4:**
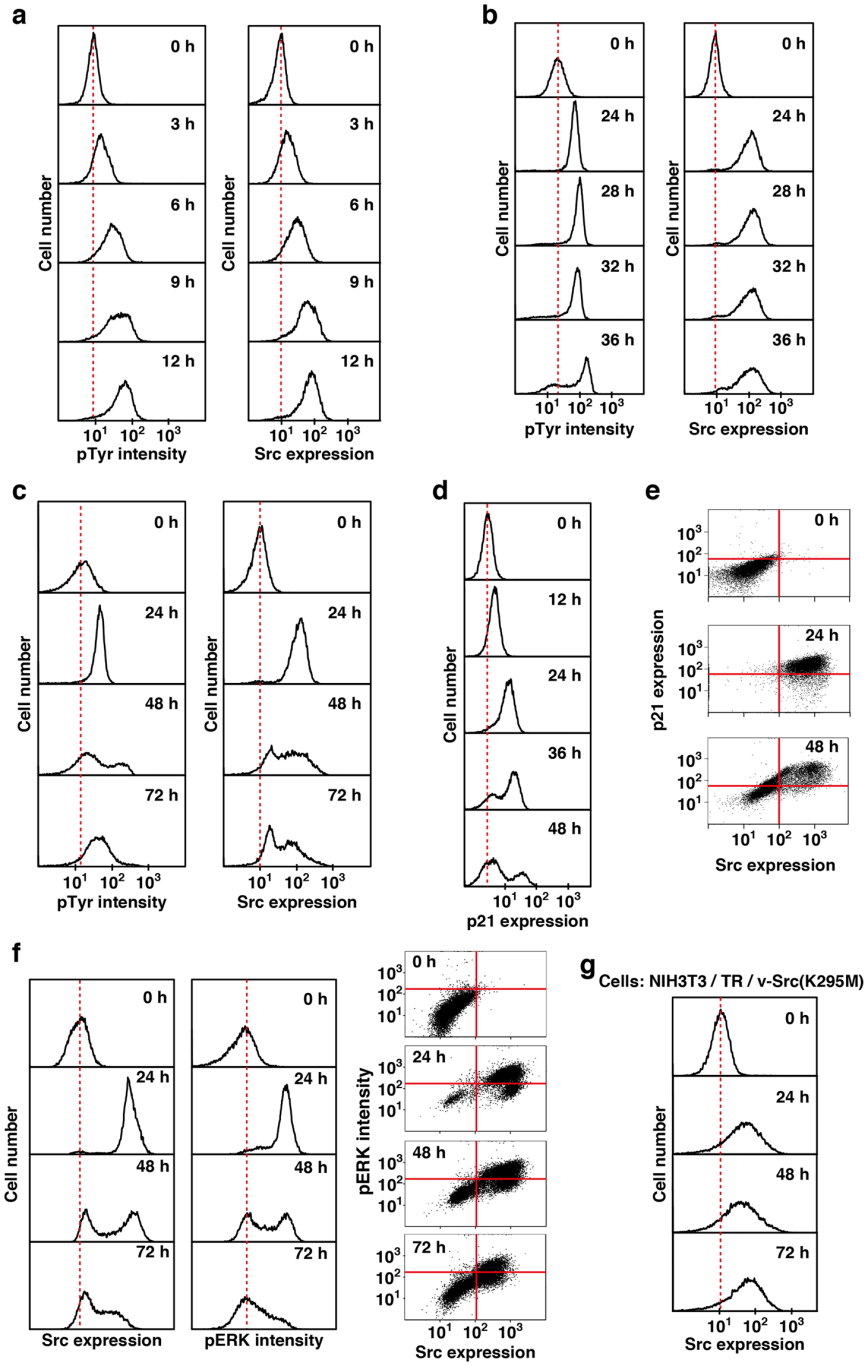
Suppression of v-Src expression in NIH3T3 cells. (**a–f**) NIH3T3/TR/v-Src-wt cells were treated with 1 µg/ml Dox for the indicated times. (**a–c**) Cells were fixed and doubly stained with anti-Src (#327) and anti-pTyr antibodies for flow cytometry. (**d**) Cells were fixed and stained with anti-p21 antibody for flow cytometry. (**e**) Cells were fixed and doubly stained with anti-Src (#327) and anti-p21 antibodies for flow cytometry. Two-dimensional histograms (Src vs p21) are presented. (**f**) Cells were fixed and doubly stained with anti-Src (#327) and anti-pERK1/2 antibodies for flow cytometry. Src and pERK histograms (left) and two-dimensional histograms (Src vs pERK) (right) are presented. (**g**) NIH3T3/TR/v-Src(K295M) cells were treated with 1 µg/ml Dox for the indicated times. Cells were fixed and stained with anti-Src (#327) antibody for flow cytometry.

Although a number of studies have shown that expression of v-Src causes oncogenic transformation of NIH3T3 cells^1,7,16,17^, our results indicate that expression of v-Src inhibits cell cycle progression despite activation of the ERK/MAPK pathway. Therefore, we hypothesized that v-Src-driven transformed cells would acquire an ability to overcome the v-Src-induced inhibitory effect on cell cycle progression. Considering that inhibition of cell cycle progression of HeLa S3 cells through p21 up-regulation is dependent on v-Src kinase activity (Figs 1,2), we kinetically examined the level of tyrosine phosphorylation of cellular proteins in NIH3T3/TR/v-Src-wt cells by flow cytometry. In the early short-term treatment with Dox (~12 h), the levels of v-Src expression and the concomitant induction of tyrosine phosphorylation were gradually increased in almost all of NIH3T3/TR/v-Src-wt cells (Fig. 4a). To our great surprise, 36 h after Dox addition, some of the cells suddenly decreased the levels of both expression of v-Src and tyrosine phosphorylation of cellular proteins (Fig. 4b). Furthermore, 48 h after Dox addition, almost half of the cells exhibited decreases in the levels of v-Src expression and tyrosine phosphorylation of cellular proteins (Fig. 4c).

Then, the level of p21 was also drastically decreased from 36 h after Dox addition as those of Src expression and cellular tyrosine phosphorylation (compare Fig. 4d with Fig. 4b,c). 2D-plot analysis revealed that the level of p21 expression was highly correlated with that of v-Src expression in NIH3T3/TR/v-Src-wt cells (Fig. 4e). The level of ERK phosphorylation was also correlated with that of v-Src expression (Fig. 4f). However, expression of v-Src(K295M) did not decrease its expression level even 72 h after Dox addition (Fig. 4g). Despite that almost all of the cells induced p21 expression along with v-Src expression soon after Dox addition, approximately half of the cells exhibited an unexpected decrease of v-Src expression and concomitant decreases of p21 expression and ERK phosphorylation until 48 h after Dox addition. These results suggest that p21 down-regulation caused by the suppression of v-Src expression is attributed to the release from v-Src-induced cell-cycle arrest.

### Re-adhesion of cells and formation of piled-up transformed foci by v-Src expression

**Figure 5:**
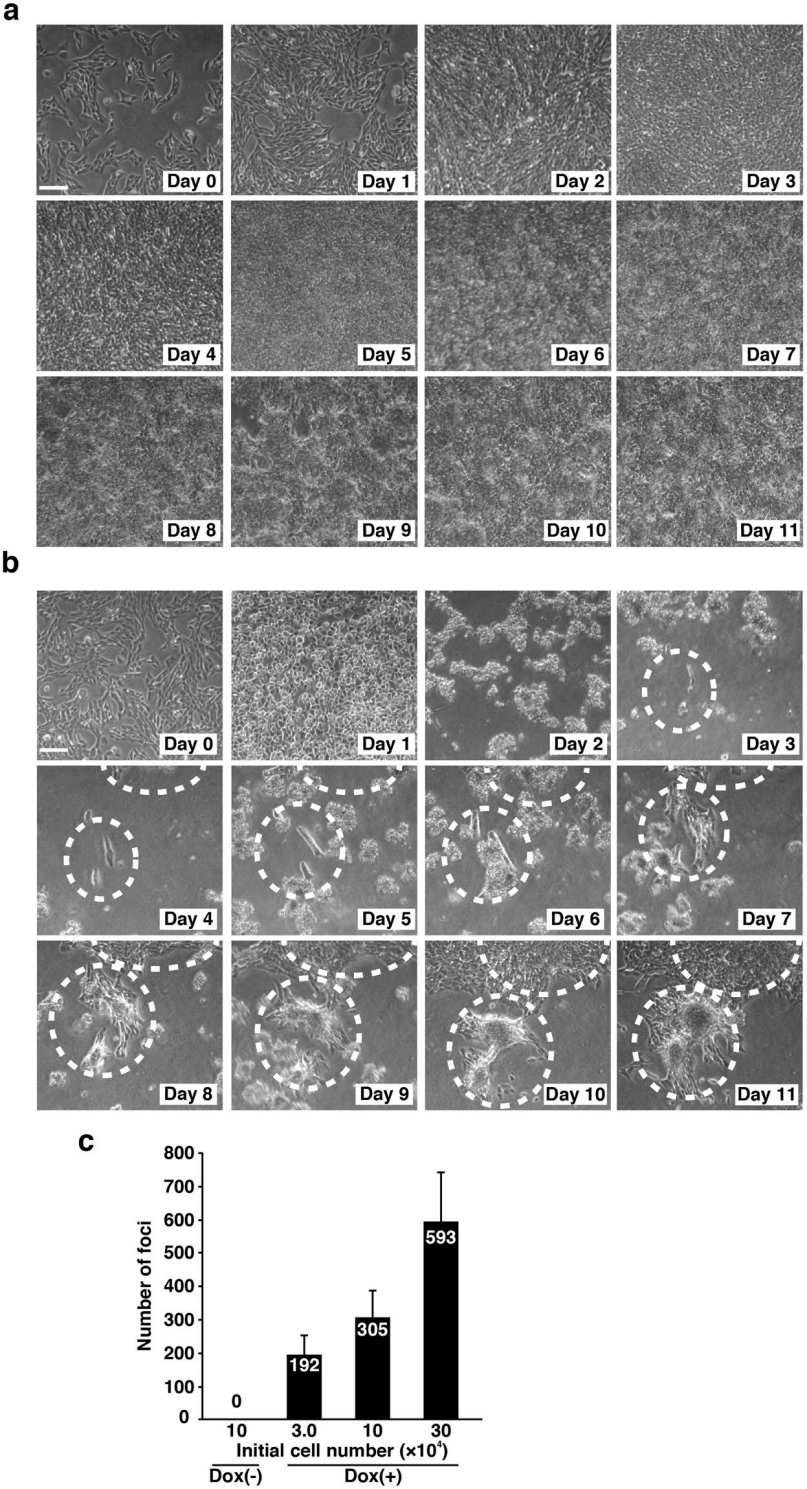
Re-adhesion of cells and formation of piled-up transformed foci by v-Src expression. (**a,b**) NIH3T3/TR/v-Src-wt cells (1 × 10^5^ cells /60-mm dish) were cultured for 1 day and treated (**a**) without or (**b**) with 1 µg/ml Dox on day 0, and the same field images were continuously observed over 11 days of culture by phase-contrast microscopy. The cells forming piled-up transformed foci are marked by dotted lines. Scale bars, 100 µm. (**c**) Colony focus formation assay. NIH3T3/TR/v-Src-wt cells at the indicated cell numbers were seeded in 60-mm dishes, cultured for 1 day, and treated with 1 µg/ml Dox for 12 days. The number of piled-up transformed foci was counted. Results represent means ± S.D. from three independent experiments.

To examine the colony-forming ability of NIH3T3/TR/v-Src-wt cells, we continuously observed NIH3T3/TR/v-Src-wt cells with or without Dox by phase-contrast microscopy. Without Dox addition, cells were able to continue to normally proliferate until confluency and did not form piled-up transformed foci (Fig. 5a). In sharp contrast, inducible expression of v-Src forced cells to become rounded on day 1 after Dox addition and to become detached from a culture dish from day 2 (Fig. 5b). From day 3, almost all of v-Src-expressing cells still remained detached, but we realized that only a limited number of the cells were capable of re-adhering to a culture dish, proliferating, and finally forming piled-up colony foci (Fig. 5b). Then, we counted the number of v-Src-induced piled-up colony foci on day 12 after Dox addition. The number of piled-up foci was correlated with the number of cells that were initially seeded (Fig. 5c). The frequencies of the appearance of v-Src-induced piled-up colony foci were approximately 0.2~0.6% among v-Src-expressing cells.

**Figure 6:**
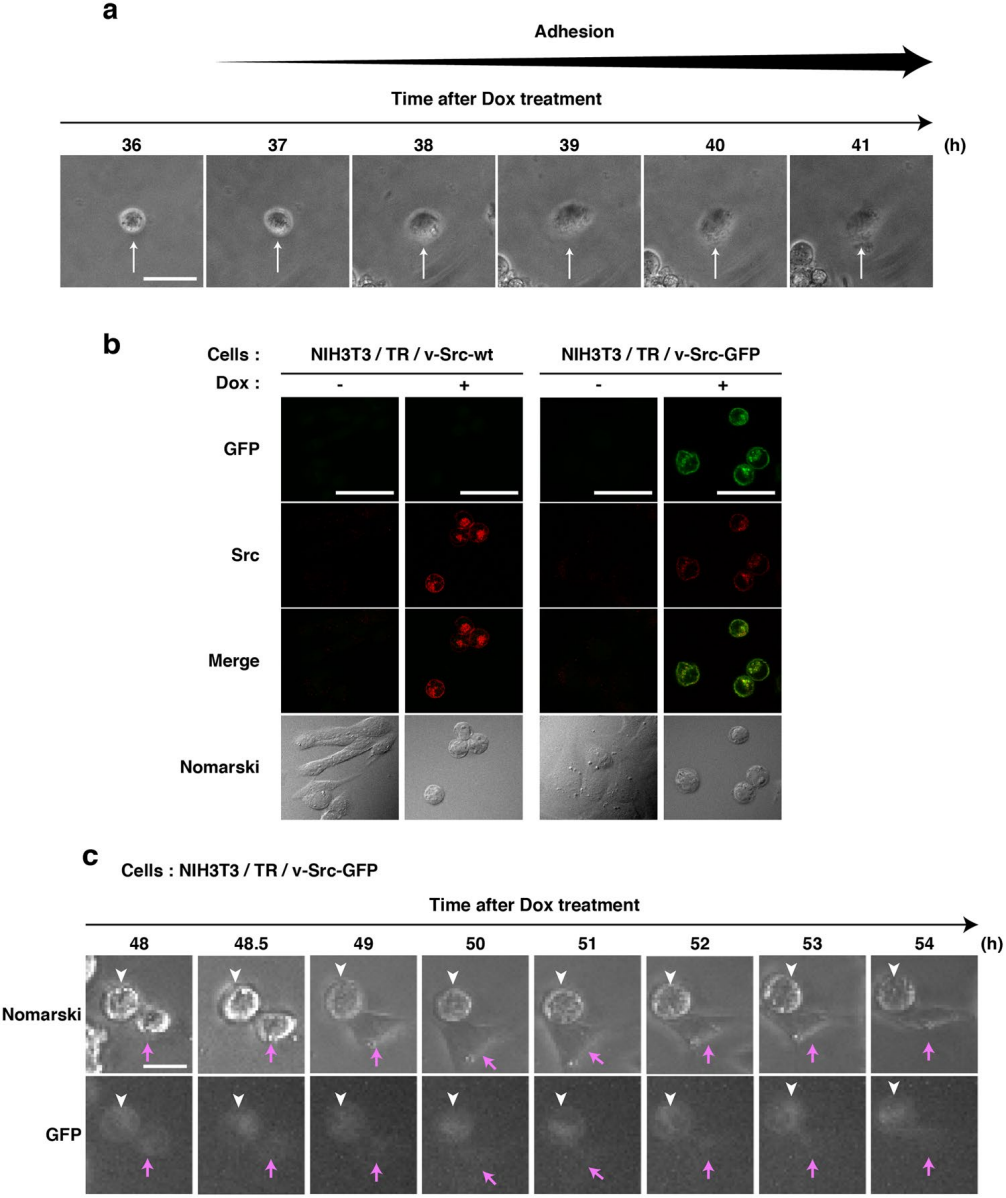
Suppression of v-Src expression in re-adherent cells. (**a**) Living NIH3T3/TR/v-Src-wt cells were monitored by time-lapse phase-contrast microscopy from 36 h after Dox addition. Arrows indicate a cell that had been detached by v-Src expression and re-adhered to a culture dish. Scale bar, 50 µm. (**b**) NIH3T3/TR/v-Src-wt cells and NIH3T3/TR/v-Src-GFP cells were cultured for 24 h with or without 1 µg/ml Dox. Expressed proteins were visualized with anti-Src (#327) antibody (red) and GFP fluorescence (green). Scale bars, 50 µm. (**c**) Living NIH3T3/TR/v-Src-GFP cells were monitored by time-lapse phase-contrast microscopy from 48 h after Dox addition. White arrowheads indicate a cell that had been detached and continuously expressed v-Src-GFP. Red arrows indicate a cell that had been detached and re-adhered to a culture dish and subsequently exhibited cell spreading while expression of v-Src-GFP was suppressed. Scale bar, 25 µm.

To monitor how v-Src-expressing cells would actually re-adhere to a culture dish, we performed time-lapse recordings of NIH3T3/TR/v-Src-wt cells from 36 h after Dox addition. Some of v-Src-expressing cells started to re-adhere to a culture dish and to exhibit cell spreading 38 h after Dox addition (Fig. 6a). Next, to monitor the expression level of v-Src in re-adherent cells, we generated NIH3T3 cells expressing inducible v-Src-GFP (v-Src-wt tagged with green fluorescent protein at its C-terminus) (NIH3T3/TR/v-Src-GFP). Like NIH3T3/TR/v-Src-wt cells, NIH3T3/TR/v-Src-GFP cells showed the high efficiency of v-Src-wt-GFP expression in a manner dependent on Dox treatment (Fig. 6b). Then, we monitored NIH3T3/TR/v-Src-GFP cells by time-lapse recordings from 48 h after Dox addition, and confirmed that v-Src-GFP-expressing cells were able to re-adhere to a culture dish. It is intriguing to note that the GFP fluorescence in v-Src-GFP-expressing cells that re-adhered and spread their morphology was found to initiate to decrease from 48.5 h after Dox addition and completely disappeared from 49 h (Fig. 6c). These results suggest that v-Src expression is not sufficient enough to allow NIH3T3 cells to form piled-up colony foci but rather subsequent suppression of v-Src expression is indispensable to the formation of piled-up colony foci, implicating that v-Src expression during at least 36 h of induction is a requisite condition for cell transformation.

### Chromosome abnormalities upon inducible expression of v-Src

**Figure 7:**
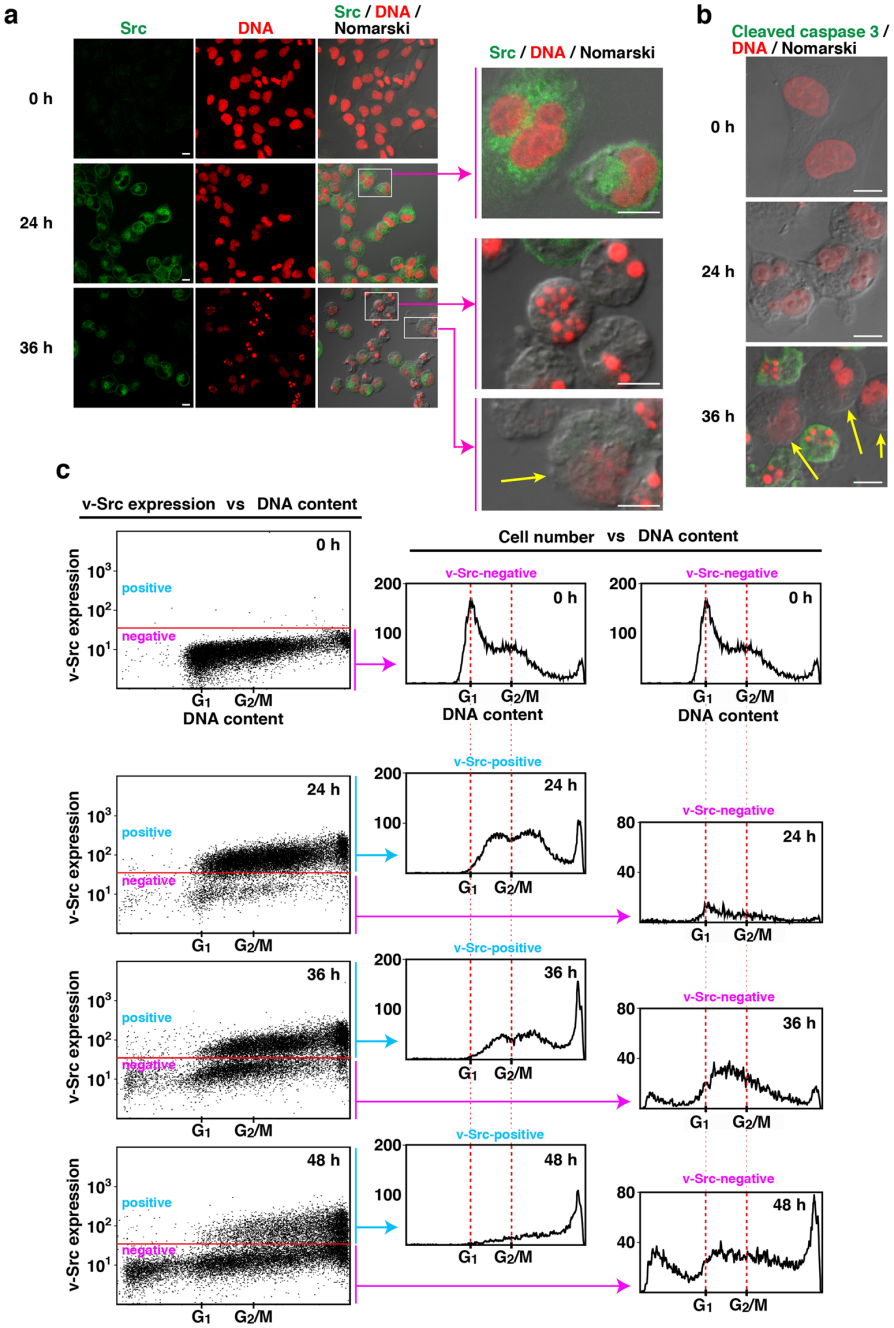
Chromosome abnormalities upon v-Src expression. (**a–c**) NIH3T3/TR/v-Src-wt cells were treated with 1 µg/ml Dox for the indicated times. (**a**) Cells were doubly stained with anti-Src (#327) antibody and PI. Magnified images of the squared areas are shown on the right. An arrow shows v-Src-negative non-adherent cell. Scale bars, 10 µm. (**b**) Cells were doubly stained with anti-cleaved caspase-3 antibody and PI. Arrows show non-apoptotic chromosome aberrant cells. Scale bars, 10 µm. (**c**) NIH3T3/TR/v-Src-wt cells were treated with 1 µg/ml Dox for the indicated times. Cells were fixed and doubly stained with anti-Src (#327) antibody and PI for flow cytometry. Two-dimensional histograms (Src vs DNA) (left) are presented. Red horizontal lines separate the region of v-Src expression (upper region) from the region of v-Src suppression (lower region). DNA histograms of the region of v-Src-expression (middle) and the region of v-Src suppression (right) are shown. The peaks of G_1_ and G_2_/M phases in v-Src-uninduced cells are marked by red vertical dotted lines. The two histograms at 0 h of Dox addition are the same control data (v-Src-uninduced cells), which enable us to easily compare differences in DNA content per cell during v-Src induction.

Our recent studies showed that v-Src expression induces chromosome missegregation/chromosomal instability, which is often caused by chromosome lagging/bridging and cytokinesis failure^12,18–21^. Thus, we kinetically visualized nuclei and DNA contents of NIH3T3/v-Src-wt cells after Dox treatment by confocal microscopy and flow cytometry, respectively (Fig. 7), and compared nuclear morphologies (Figs 7a,b) with DNA histograms (Fig. 7c). Note that before Dox treatment (0 h), the expression of endogenous c-Src was only seen in NIH3T3/TR/v-Src-wt cells, and the peak positions of G_1_ and G_2_/M phases were marked. After 24 h of v-Src induction, almost all of the cells were still viable and showed deformed nuclei (Fig. 7a), including lobulated nuclei, binuclei and multinuclei, with aberrantly increased DNA contents [Fig. 7c, v-Src-positive (24 h)]. After 36 h of v-Src induction, around half of the cells suppressed v-Src expression [Fig. 7a (36 h)] and a fraction of the cells suppressing v-Src expression underwent apoptosis with typical chromosome fragmentation/condensation and caspase activation (the appearance of cleaved caspase-3) [Fig. 7b (36 h)], which corresponds to the cells having chromosome abnormalities, i.e., a broad range of DNA contents from subG_1_ to polyploidy [Fig. 7c, v-Src-negative (36 h); compare v-Src-negative (36 h) with v-Src-negative (0 h) in Fig. 7c]. Some of the viable cells that lost v-Src expression showed small dotted aggregates of DNA and deformed nuclei [Fig. 7a,b, arrows (36 h)], suggesting that this cell population contains cells capable of forming piled-up transformed colony foci. These results suggest that v-Src expression induces chromosome abnormalities in NIH3T3 cells until 24 h after v-Src induction and subsequent suppression of v-Src expression diversifies the DNA content per cell. Because v-Src induction for 24 h brought about deformed nuclei with aberrantly increased DNA contents, a variety of chromosome abnormalities may happen in all of v-Src-expressing cells, leading to a cause of suppression of v-Src expression.

## Discussion

In the present study, we utilize HeLa S3 and NIH3T3 cells exhibiting extremely high expression efficiencies of tetracycline-inducible v-Src, and show that v-Src expression induces not only ERK activation but also p21 up-regulation, resulting in cell cycle arrest. Since we demonstrated that cell proliferation is inhibited by v-Src expression in a manner responsive to a wide range of Dox concentrations (30 pg/ml ~1 µg/ml) in three different cell types, i.e., HeLa S3 cervical carcinoma cells, HCT116 colorectal carcinoma cells, and NIH3T3 fibroblast cells^12^, these results suggest that cell cycle arrest seen upon v-Src expression is indeed an unambiguous and intrinsic phenotype of v-Src.

**Figure 8:**
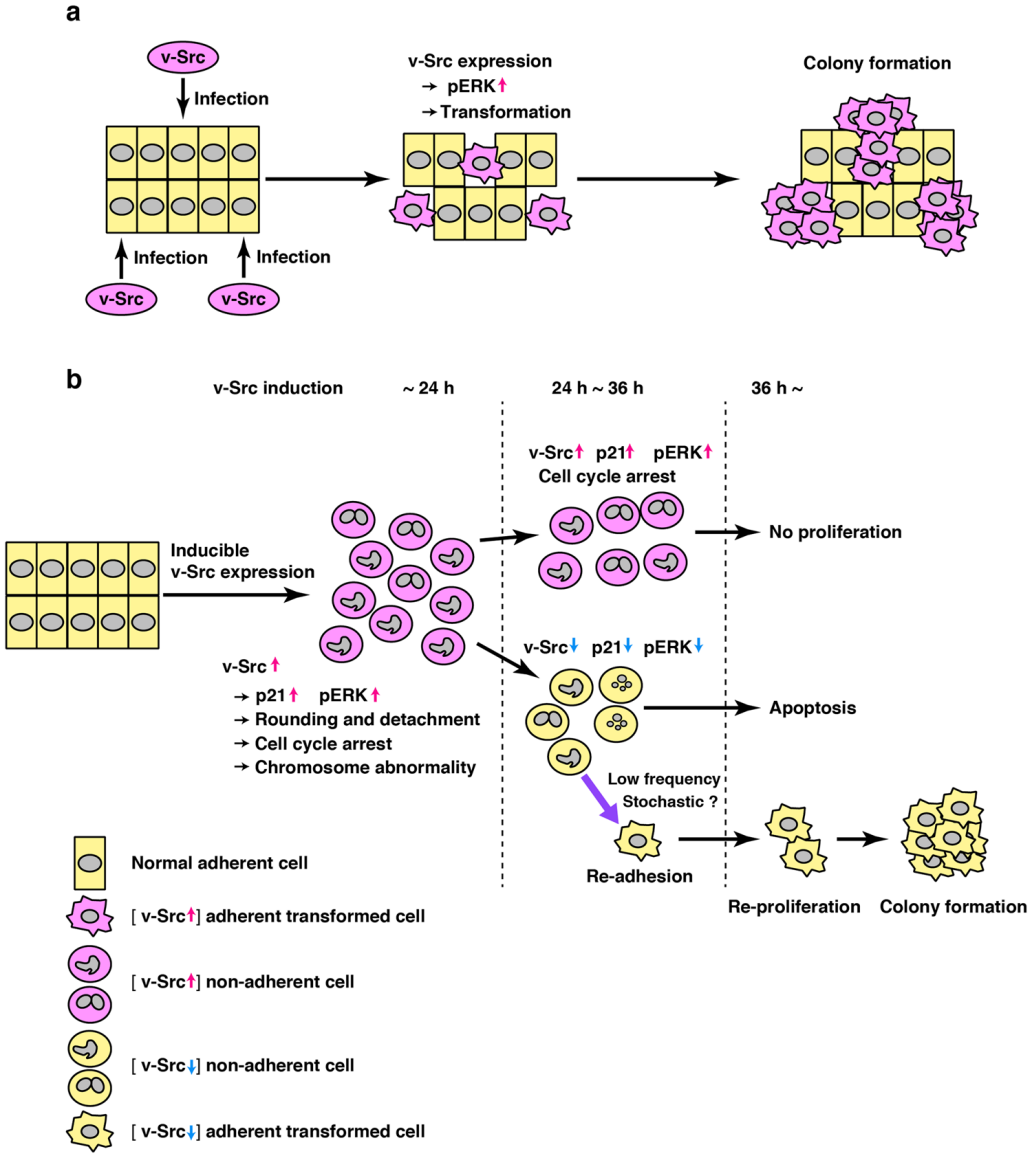
A renewed model of v-Src-induced tumorigenesis. (**a**) The commonly accepted concept of v-Src-induced tumorigenesis. Expression of v-Src induces activation of downstream signaling pathways, including the ERK/MAPK pathway. Continuous expression of v-Src forces cells to proliferate uncontrollably, which leads to oncogenic transformation. (**b**) Our renewed model of v-Src-induced tumorigenesis. During the first 24 h after v-Src induction, all of v-Src-expressing cells exhibit (i) up-regulation of pERK and p21, (ii) cell rounding and detachment, (iii) cell cycle arrest, and (iv) chromosome abnormalities. From 24 h to 36 h after v-Src induction, v-Src-expressing cells are unable to proliferate due to continuous up-regulation of p21. However, approximately half of v-Src-expressing cells happen to suppress the level of v-Src expression, resulting in decreases of p21 and pERK to baseline levels. A limited number of cells suppressing inducible v-Src can only gain the ability to re-adhere to a culture dish, re-proliferate vigorously and finally form transformed colonies.

However, we verify that v-Src expression undoubtedly brings about the formation of piled-up colonies of NIH3T3 cells. This paradox can be resolved by our present results that unexpected suppression of v-Src expression leads to p21 down-regulation besides ERK inactivation. Among cells that suppress v-Src expression, a limited number of cells can only gain the ability to re-adhere to a culture dish, re-proliferate, and form piled-up colonies. Notably, v-Src expression induces chromosome abnormalities and the subsequent suppression of v-Src expression brings about a broad range of the DNA content per cell. Accordingly, v-Src-induced chromosome abnormalities may account for suppression of v-Src expression and subsequent induction of colony formation. In other words, the onset of v-Src-induced genetic instability could happen at a very low frequency for deserving cell transformation. Our findings are profoundly different from the commonly accepted current concept that v-Src continuously stimulates growth signaling by tyrosine phosphorylation to promote tumorigenesis (Fig. 8a). We would inspire a renewal of the current concept to support our novel findings (Fig. 8b).

Historically, v-Src-induced colony formation assays have been carried out by infection with virions/retroviral vectors or transfection with v-Src expression vectors using chemical reagents. It has been believed that the more efficient v-Src expression is, the more colonies one will see. However, if v-Src is expressed in more than 10% of cells, all of v-Src-expressing cells would proliferate and overflow a culture dish so that individual colonies could not be counted due to being overlapped and indistinguishable. In fact, much of the literature indicates the low frequencies of the appearance of v-Src-induced piled-up colonies^9–11^, implying that the low frequencies of colony formation might be somehow considered as a result of low gene transfer rates. However, in our experiments, even though almost 100% efficiency in v-Src expression is achieved, the number of observed piled-up colonies is only 0.2~0.6% of the initial cell number (Fig. 5c). When we transfected parental NIH3T3 cells with v-Src, the transfection efficiency was 19 ± 5% after 24 h of transfection and the frequency of the formation of piled-up colony foci on day 18 after transfection was 0.01% among v-Src-expressing cells. This low efficiency was comparable to those of Dox-induced v-Src-expressing NIH3T3/TR/v-Src-wt cells. Importantly, these transformed colonies are originated from the cells in which initially expressed v-Src was suppressed (Figs 5,6,7). When we picked up several transformed colonies, individual colonies were found to continue to suppress v-Src expression, indicating that v-Src expression in the early phase of induction is indispensable to the formation of piled-up colonies.

The ERK/MAPK pathway is one of the major signaling events downstream of Src for growth stimulation^13^. Nonetheless, suppression of v-Src expression results in a drastic decrease in the level of activated ERK (Fig. 4f), suggesting that v-Src-mediated re-proliferation and colony formation no longer require v-Src-mediated continuous activation of the growth signaling pathways. Intriguingly, v-Src expression until 24 h after v-Src induction triggers chromosome abnormalities in all of v-Src-expressing cells, and the subsequent suppression of v-Src expression results in chromosome abnormalities with a broad range of DNA contents distributing from lower to higher ploidy levels (Fig. 7c). It should be emphasized that almost all of the cells with high expression of v-Src become rounded and detached from a culture dish [Fig. 5b (day 2), Fig. 7a,b (24 h, 36 h)]. This morphological change does not occur unless v-Src is highly expressed [Fig. 5a, Fig. 7a,b (0 h)]. We treated NIH3T3/TR/v-Src-wt cells (1 × 10^5^ initial cells) with 1 µg/ml Dox for 48 h, collected detached cells, re-seeded them onto a culture dish, and observed colony formation for 10~20 days. 124 ± 62 piled-up colony foci were formed in three independent experiments. These results further substantiate that a fraction of the cells highly expressing v-Src are capable of proliferating and forming piled-up colony foci when v-Src expression is suppressed. Owing to extremely low frequencies of the appearance of v-Src-induced transformed colonies, v-Src-induced chromosome abnormalities would cause oncogenic transformation possibly through stochastic genetic alterations (Fig. 8b).

The landmark paper by Temin and Rubin described that the number of chromosomes found in less than l-month-old transformed cells by infection with Rous sarcoma virus (Rous sarcoma cells) and in normal chick fibroblasts was the same and the mode was in the middle seventies for both types of cells and the spread was similar^22^. However, we noticed their misinterpretation of the data. In fact, their published data evidently indicate that the number of chromosomes in Rous sarcoma cells was distributed in a broad range of number per cell, compared with that of normal chick fibroblasts. The paper also described that, after more prolonged culture of the Rous sarcoma cells at a time when many giants were present, over half of the cells were polyploid. Therefore, from the very beginning of the study on transformation by Rous sarcoma virus, one must have unconsciously seen that v-Src expression by Rous sarcoma virus infection brings about chromosome abnormalities.

The levels of CDK inhibitor proteins, including p21, are increased by activation of the p53 tumor suppressor protein in the DNA damage response^15^. p21 inhibits the activities of CDK2 in G_1_ phase and CDK1 in G_2_/M phase, leading to cell cycle arrest at G_1_ and G_2_ phase^23^. Although c-Src phosphorylated p27/Kip1, another CDK inhibitor protein, and inhibited its activity^24^, the relationship between Src and p21 is poorly understood. We show that v-Src up-regulates the levels of p21 in a manner dependent on v-Src kinase activity (Figs 2,4). Given that ubiquitination and degradation of p53 are increased in HeLa cells^25^, it is likely that v-Src-mediated up-regulation of p21 may be independent of p53 activity.

Constitutively active c-Src is thought to induce oncogenic transformation through increased cell proliferation^26^ (Fig. 8a). However, it is reported that increases in c-Src levels and activity do not promote cell proliferation^27^. When NIH3T3/TR/c-Src-HA(Y530F) cells (Supplementary Fig. S7a,b) were compared with NIH3T3/TR/v-Src-wt cells, c-Src-HA(Y530F) expression modestly increased tyrosine phosphorylation of cellular proteins and slowly suppressed Src expression (Supplementary Fig. S7c,d). Even though the kinase activity of c-Src-HA(Y530F) is lower than that of v-Src (Supplementary Fig. S7c), a limited number of c-Src-HA(Y530F)-suppressed cells were enabled to re-adhere to a culture dish, re-proliferate, and form piled-up colonies (Supplementary Fig. S8). Essentially, c-Src-HA(Y530F)-expressing cells can behave as v-Src-expressing cells but in a slow and modest manner.

Although Src substrates have been extensively hunted so far^5,6,28^, we showed that Src-family members are localized to the nucleus besides the plasma membrane^29–31^ and revealed novel roles for Src-mediated tyrosine phosphorylation of chromosome-associated proteins^32–34^. Given that chromosome abnormalities happen until 24 h after v-Src induction (Figs 4,6,7), we hypothesize that some chromosome-associated proteins that are normally away from tyrosine phosphorylation may be aberrantly phosphorylated by v-Src in this period. Identification of such proteins will uncover the mechanism of v-Src-mediated transformation.

In conclusion, we show that continuous expression of v-Src induces cell cycle arrest. Moreover, v-Src-induced chromosome abnormalities may stochastically cause oncogenic transformation in NIH3T3 cells. Thus, we propose to fundamentally reconsider the mechanism of Src-induced tumorigenesis.

## Methods

### Plasmids

To construct the pcDNA4/TO/v-Src-wt and pcDNA4/TO/v-Src(K295M) vectors for inducible expression of wild-type v-Src (v-Src-wt) and kinase-dead v-Src [v-Src(K295M)], the *Hind*III-*Xho*I fragment of pcDNA3/v-Src-wt and pcDNA3/v-Src(K295M) (gifted by H. Ohnishi^35^) were subcloned into the *Hind*III-*Xho*I site of pcDNA4/TO/neoR^12,36^. The green fluorescent protein (GFP)-fused v-Src fragment was created by PCR using the sense primer 5′-CCGCTAGTAGGTACCATGGGGAGTAGCAAGAGCAAG-3′ and the antisense primer 5′-CCTACTAGCACCGGTCGCTCAGCGACCTCCAACACAC-3′. The *KpnI-AgeI* fragment of the PCR products were introduced into the *KpnI-AgeI* site of pcDNA4/TO/GFP/neoR.

### Cells and transfection

HeLa S3 (Japanese Collection of Research Bioresources, Osaka) and NIH3T3 cells were cultured in Iscove’s modified Dulbecco’s medium (IMDM) containing 1% fetal bovine serum (FBS) and 4% bovine serum (BS). Transfection was performed using linear polyethylenimine (25 kDa; Polysciences)^37^. Stable cell lines for tetracycline-inducible v-Src expression in HeLa S3 and NIH3T3 were generated as reported previously^12^. To generate cell lines for tetracycline-inducible v-Src(K295M) expression in HeLa S3 cells, tetracycline repressor (TR)-expressing HeLa S3 cells (HeLa S3/TR clone A3f5) were transfected with pcDNA4/TO/v-Src(K295M) and selected in 200 µg/ml G418 (Wako Pure Chemicals, Osaka). To generate cell lines for tetracycline-inducible v-Src(K295M) or v-Src-GFP expression in NIH3T3 cells, TR-expressing NIH3T3 cells were transfected with pcDNA4/TO/v-Src(K295M) or pcDNA4/TO/v-Src-GFP and selected in 125 μg/ml or 250 μg/ml G418.

### Cell synchronization

To synchronize cells at the G_1_/S boundary, a double thymidine block was performed as described^20,38,39^. In brief, cells treated with 4 mM thymidine for 16 h were incubated in thymidine-free complete medium for 9 h, and the cells were further treated with 4 mM thymidine for 16 h.

### Antibodies

The following mouse monoclonal antibodies were used: v-Src (#327, Calbiochem), Src (GD11, Millipore), phosphotyrosine (pTyr, 4G10, Upstate Biotechnology), and actin (C4, Millipore). The following rabbit monoclonal antibodies were used: phospho-p44/42 MAPK (ERK1/2) (Thr202/Tyr204) (D13.14.4E, Cell Signaling Technology), and p21 Waf1/Cip1 (12D1, Cell Signaling Technology). The following rabbit polyclonal antibodies were used: c-Src (N-16, Santa Cruz Biotechnology), ERK2 (C-14, Santa Cruz Biotechnology), Src[pY^416^] (phospho-Src family, Cell Signaling Technology), p16 (C-20, Santa Cruz Biotechnology), p21 (C-19, Santa Cruz Biotechnology) and Cleaved Caspase-3 (Asp175, Cell Signaling Technology). The following rat monoclonal antibody was used: α-tubulin (MCA78G, Serotec). Horseradish peroxidase-F(ab’)_2_ fragments of anti-mouse IgG antibody (GE Healthcare), anti-rabbit IgG antibody (Cell LAB), and anti-rat IgG antibody (GE Healthcare) were used. Alexa Fluor 488-donkey-anti-mouse IgG and Alexa Fluor 647-goat-anti-mouse IgG secondary antibodies were obtained from Invitrogen.

### Immunofluorescence microscopy

Immunofluorescence staining was performed as described^12,40–43^. In brief, cells were fixed in phosphate-buffered saline (PBS)-containing 4% paraformaldehyde for 20 min. Then, fixed cells were blocked in PBS containing 0.1% saponin and 3% bovine serum albumin, and sequentially incubated with a primary and a secondary antibody for 1 h each. For DNA staining, cells were subsequently treated with 200 µg/ml RNase A for 30 min and 20 µg/ml propidium iodide (PI), an intercalating agent, for 30 min. Stained cells were mounted with Antifade reagent. Confocal and Nomarski differential-interference-contrast images were obtained using an LSM510 laser-scanning microscope (Zeiss) and an FV500 laser scanning microscope (Olympus). Composite figures were prepared using GIMP version 2.6.2 and Illustrator CS6 software (Adobe).

### Flow cytometry

Flow cytometric analysis was performed as described^12,38,39^. Cells were detached by trypsinization for 3~5 min, fixed with 1.5% paraformaldehyde at room temperature for 30 min, and then permeabilized with 70% ethanol for at least 1 h at −30 °C. After washing twice with PBS containing 3% BS and 0.1% Tween 20, cells were reacted with primary antibodies for 1 h at room temperature, and then stained with secondary antibodies for 1 h. For staining DNA, cells were incubated with 200 µg/ml RNase A and 50 µg/ml PI at 37 °C for 30 min. Each sample was analyzed by flow cytometry using a Guava easyCyte (Millipore) equipped with a 488-nm blue laser and a 640-nm red laser using liner amplification. Dead cells and debris were excluded by gating on forward scatter and pulse-width profiles. Acquired data were analyzed using Flowing Software version 2.5.0 (Perttu Terho, Centre for Biotechnology, Turku, Finland).

### Western blotting

Cell lysates were prepared in SDS-sample buffer were separated by SDS-PAGE and electrotransferred onto polyvinylidene difluoride membranes (PVDF, Millipore). Immunodetection was performed as reported previously^20,34,36,43^. Sequential reprobing of membranes with a variety of antibodies was performed after the complete removal of primary and secondary antibodies from membranes in stripping buffer or inactivation of horseradish peroxidase by 0.1% NaN_3_, according to the manufacturer’s instructions. Results were analyzed using an image analyzer ChemiDoc XRSplus (Bio-Rad). Intensity of chemiluminescence was measured using the Quantity One software (Bio-Rad).

### Colony focus formation assay

NIH3T3/TR/v-Src-wt cells were seeded in 60-mm dishes and cultured for the indicated times with or without 1 µg/ml Dox. The culture medium was changed daily by replacing with fresh medium that was supplemented with or without Dox after cells were cultured for 4 or 5 days.

### Time-lapse recording

NIH3T3/TR/v-Src-wt cells and NIH3T3/TR/v-Src-GFP cells were treated with 1 µg/ml Dox for 36 hr. Subsequently, the cells were cultured in pre-warmed fresh medium supplemented with 1 µg/ml Dox and 20 mM HEPES-NaOH (pH 7.3). The culture dish was placed on a 37 °C preheated stage of an inverted Zeiss Axiovert S100 microscope with a 20 × 0.50 N.A. objective, and the phase contrast images and the fluorescence of GFP were monitored. After the time-lapse recordings, the images were analyzed using ImageJ software (NIH, USA).

## Electronic supplementary material

Supplementary Figures

## References

[1] Thomas SM, Brugge JS (1997). Cellular functions regulated by Src family kinases. Annu. Rev. Cell Dev. Biol..

[2] Hubbard SR, Till JH (2000). Protein tyrosine kinase structure and function. Annu. Rev. Biochem..

[3] Hunter T (2009). Tyrosine phosphorylation: thirty years and counting. Curr. Opin. Cell Biol..

[4] Irby RB, Yeatman TJ (2000). Role of Src expression and activation in human cancer. Oncogene.

[5] Yeatman TJ (2004). A renaissance for Src. Nat. Rev. Cancer.

[6] Brown MT, Cooper JA (1996). Regulation, substrates and functions of src. Biochim. Biophys. Acta.

[7] Martin GS (2001). The hunting of the Src. Nat. Rev. Mol. Cell Biol..

[8] Vogt PK (2012). Retroviral oncogenes: a historical primer. Nat. Rev. Cancer.

[9] Shalloway D, Coussens PM, Yaciuk P (1984). Overexpression of the c-src protein does not induce transformation of NIH 3T3 cells. Proc. Natl. Acad. Sci. USA.

[10] Bromberg JF, Horvath CM, Besser D, Lathem WW, Darnell JE (1998). Stat3 activation is required for cellular transformation by v-src. Mol. Cell. Biol..

[11] Kazansky AV, Rosen JM (2001). Signal transducers and activators of transcription 5B potentiates v-Src-mediated transformation of NIH-3T3 cells. Cell growth Differ..

[12] Soeda S (2013). v-Src causes delocalization of Mklp1, Aurora B, and INCENP from the spindle midzone during cytokinesis failure. Exp. Cell Res..

[13] Frame MC (2002). Src in cancer: deregulation and consequences for cell behaviour. Biochim. Biophys. Acta.

[14] Ishizawar R, Parsons SJ (2004). c-Src and cooperating partners in human cancer. Cancer Cell.

[15] Besson A, Dowdy SF, Roberts JM (2008). CDK inhibitors: cell cycle regulators and beyond. Dev. Cell.

[16] Shalloway D, Johnson PJ, Freed E, Coulter D, Flood WA (1987). Transformation of NIH 3T3 cells by cotransfection with nuclear oncogenes. Mol. Cell. Biol..

[17] Reddy S, Yaciuk P, Kmiecik TE, Coussens PM, Shalloway D (1988). v-src mutations outside the carboxyl-coding region are not sufficient to fully activate transformation by pp60c-src in NIH 3T3 cells. Mol. Cell. Biol..

[18] Ikeuchi M (2016). v-Src causes chromosome bridges through generating DNA damage. Int. J. Mol. Sci..

[19] Kakae K (2017). v-Src-induced nuclear localization of YAP is involved in multipolar spindle formation in tetraploid cells. Cell. Signal..

[20] Honda T (2016). Protective role for lipid modifications of Src-family kinases against chromosome missegregation. Sci. Rep..

[21] Nakayama Y, Soeda S, Ikeuchi M, Kakae K, Yamaguchi N (2017). Cytokinesis failure leading to chromosome instability in v-Src-induced oncogenesis. Int. J. Mol. Sci..

[22] Temin HM, Rubin H (1958). Characteristics of an assay for Rous sarcoma virus and Rous sarcoma cells in tissue culture. Virology.

[23] Abbas T, Dutta A (2009). p21 in cancer: intricate networks and multiple activities. Nat. Rev. Cancer.

[24] Chu I (2007). p27 phosphorylation by Src regulates inhibition of cyclin E-Cdk2. Cell.

[25] Scheffner M, Huibregtse JM, Vierstra RD, Howley PM (1993). The HPV-16 E6 and EG-AP complex functions as a ubiquitin-protein ligase in the ubiquitination of p53. Cell.

[26] Kim LC, Song L, Haura EB (2009). Src kinases as therapeutic targets for cancer. Nat. Rev. Clin. Oncol..

[27] Welman A (2006). Increases in c-Src expression level and activity do not promote the growth of human colorectal carcinoma cells *in vitro* and *in vivo*. Neoplasia.

[28] Reynolds AB (2010). SRChing for the substrates of Src. Oncogene.

[29] Yamaguchi N (2001). Overexpression of the Csk homologous kinase (Chk tyrosine kinase) induces multinucleation: a possible role for chromosome-associated Chk in chromosome dynamics. J. Cell Sci..

[30] Ikeda K (2008). Nuclear localization of Lyn tyrosine kinase mediated by inhibition of its kinase activity. Exp. Cell Res..

[31] Takahashi A (2009). Nuclear localization of Src-family tyrosine kinases is required for growth factor-induced euchromatinization. Exp. Cell Res..

[32] Kubota S (2013). Phosphorylation of KRAB-associated protein 1 (KAP1) at Tyr-449, Tyr-458, and Tyr-517 by nuclear tyrosine kinases inhibits the association of KAP1 and heterochromatin protein 1α (HP1α) with heterochromatin. J. Biol. Chem..

[33] Kubota S (2015). Role for tyrosine phosphorylation of A-kinase anchoring protein 8 (AKAP8) in its dissociation from chromatin and the nuclear matrix. J. Biol. Chem..

[34] Morii M (2017). Src acts as an effector for Ku70-dependent suppression of apoptosis through phosphorylation of Ku70 at Tyr-530. J. Biol. Chem..

[35] Ohnishi H (2001). A src family tyrosine kinase inhibits neurotransmitter release from neuronal cells. Proc. Natl. Acad. Sci. USA.

[36] Nakayama Y (2009). Bleomycin-induced over-replication involves sustained inhibition of mitotic entry through the ATM/ATR pathway. Exp. Cell Res..

[37] Fukumoto Y (2010). Cost-effective gene transfection by DNA compaction at pH 4.0 using acidified, long shelf-life polyethylenimine. Cytotechnology.

[38] Kubota S (2014). Activation of the prereplication complex is blocked by mimosine through reactive oxygen species-activated ataxia telangiectasia mutated (ATM) protein without DNA damage. J. Biol. Chem..

[39] Hasegawa H (2014). Cdk1-mediated phosphorylation of human ATF7 at Thr-51 and Thr-53 promotes cell-cycle progression into M phase. PLoS One.

[40] Yamaguchi N, Fukuda MN (1995). Golgi retention mechanism of ß-1,4-galactosyltransferase: membrane-spanning domain-dependent homodimerization and association with α- and ß-tubulins. J. Biol. Chem..

[41] Kasahara K (2004). Trafficking of Lyn through the Golgi caveolin involves the charged residues on αE and αI helices in the kinase domain. J. Cell Biol..

[42] Sato I (2009). Differential trafficking of Src, Lyn, Yes and Fyn is specified by the state of palmitoylation in the SH4 domain. J. Cell Sci..

[43] Obata Y (2010). The Lyn kinase C-lobe mediates Golgi export of Lyn through conformation-dependent ACSL3 association. J. Cell Sci..

