# Supplementary material for: v-Src-driven transformation is due to chromosome abnormalities but not Src-mediated growth signaling
Source: Sci Rep. 2018 Jan 18;8:1063. doi: 10.1038/s41598-018-19599-1 (PMC5773541; doi:10.1038/s41598-018-19599-1)
Supplement: Supplementary file 1 — Supplementary Figures [file 41598_2018_19599_MOESM1_ESM.pdf]

Supplementary Information

v-Src-driven transformation is due to chromosome abnormalities but not Src-mediated growth signaling

Takuya Honda[1], Mariko Morii[1], Yuji Nakayama[2], Ko Suzuki[1], Noritaka Yamaguchi[1], and Naoto Yamaguchi[1,*]

[1]Laboratory of Molecular Cell Biology, Graduate School of Pharmaceutical Sciences, Chiba University, Chiba 260-8675, Japan

[2]Department of Biochemistry and Molecular Biology, Kyoto Pharmaceutical University, Kyoto 607-8414, Japan

*To whom correspondence should be addressed:
Naoto Yamaguchi, Ph.D.
Laboratory of Molecular Cell Biology, Graduate School of Pharmaceutical Sciences, Chiba University, Inohana 1-8-1, Chuo-ku, Chiba 260-8675, Japan.
Phone & Fax: +81-43-226-2868. E-mail: nyama@faculty.chiba-u.jp

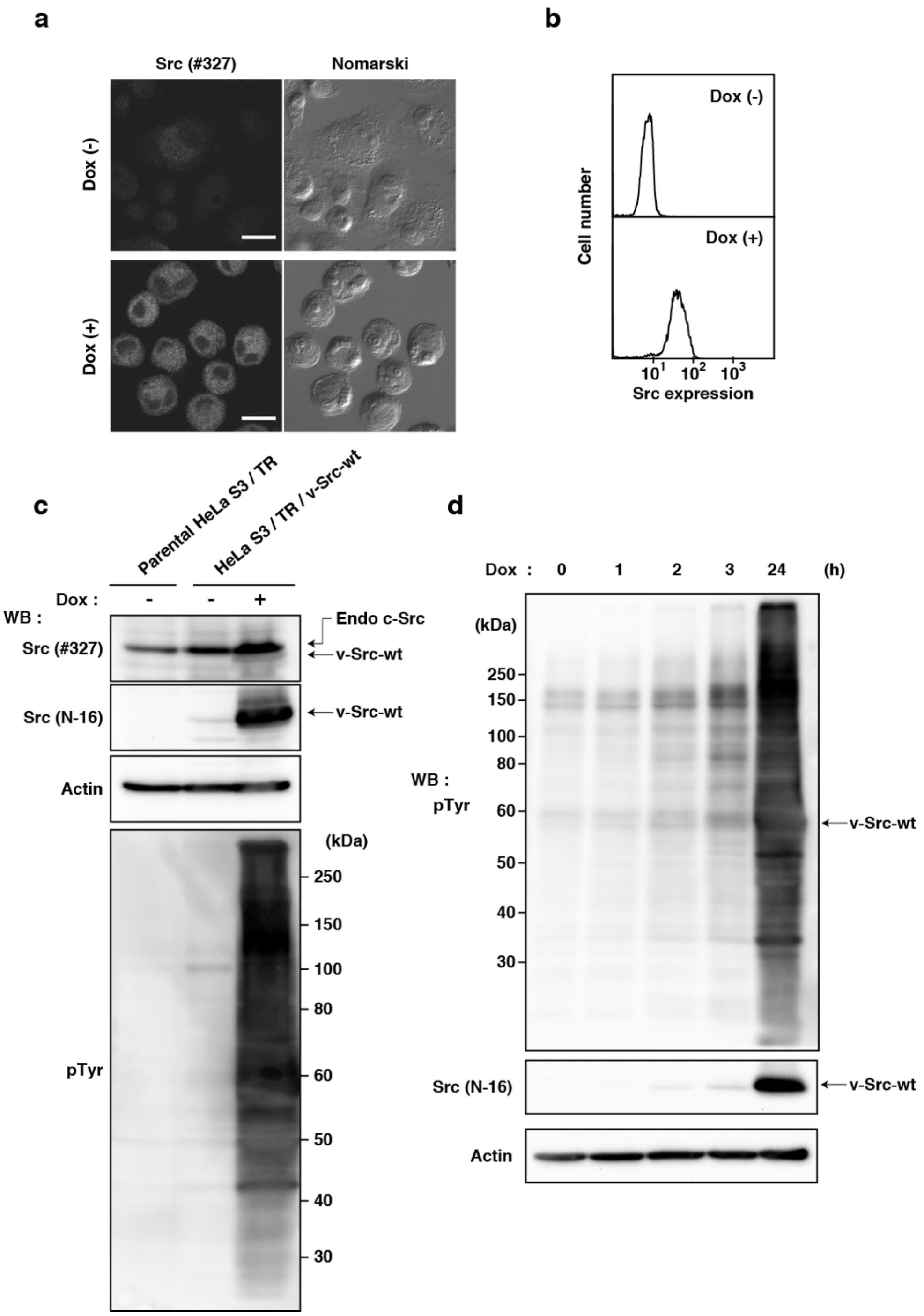

Supplementary Fig. S1. Extremely high expression efficiency of inducible v-Src.

(a, b) HeLa S3 cells expressing inducible v-Src-wt (HeLa S3/TR/v-Src-wt) were cultured for 24 h with or without 1 µg/ml doxycycline (Dox). Cells were fixed and immunostained with anti-Src (#327) antibody. **(a)** Confocal microscopic images and **(b)** flow cytometric histograms were shown. Scale bars, 20 µm. **(c)** Parental HeLa S3/TR and HeLa S3/TR/v-Src-wt cells were cultured for 24 h with or without 1 µg/ml Dox. Whole cell lysates were analyzed by Western blotting (WB) using anti-Src (#327), anti-Src (N-16), anti-actin (loading control), and anti-phosphotyrosine (pTyr) antibodies. Endo c-Src stands for endogenous c-Src. **(d)** HeLa S3/TR/v-Src-wt cells were cultured with 1 µg/ml Dox for the indicated times. Whole cell lysates were analyzed by Western blotting using anti-pTyr, anti-Src (N-16), and anti-actin antibodies.

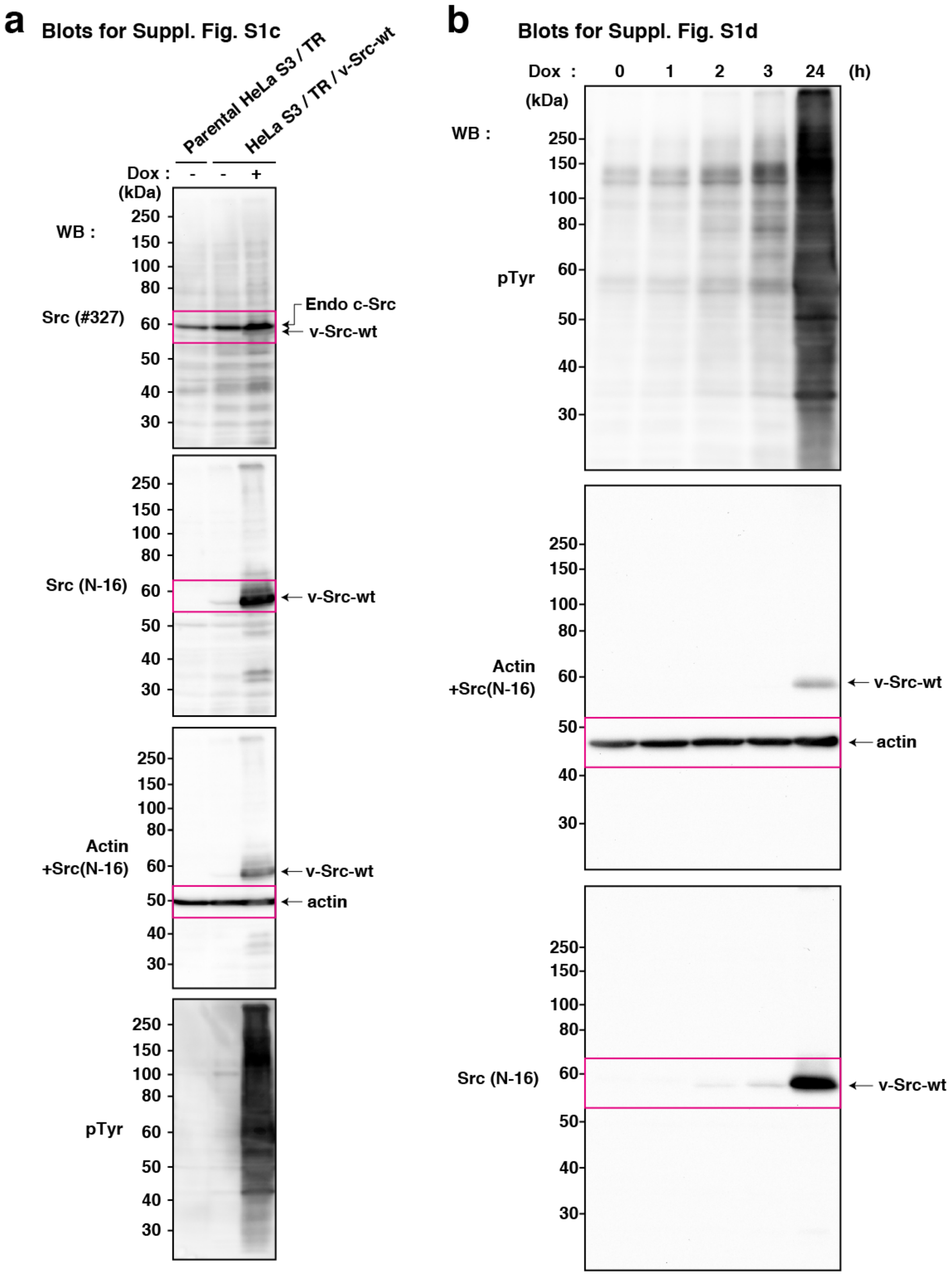

Supplementary Figure S2. Full-length blots. **(a)** Full-length blots for Supplementary Fig. S1c. **(b)** Full-length blots for Supplementary Fig. S1d.

a

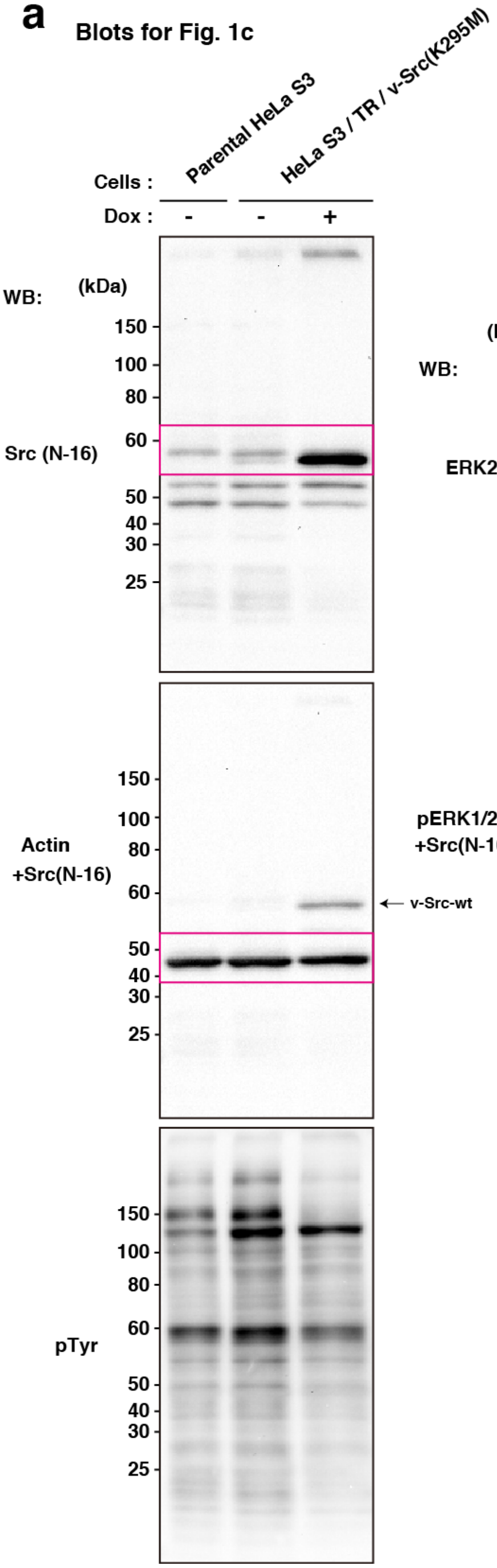

b

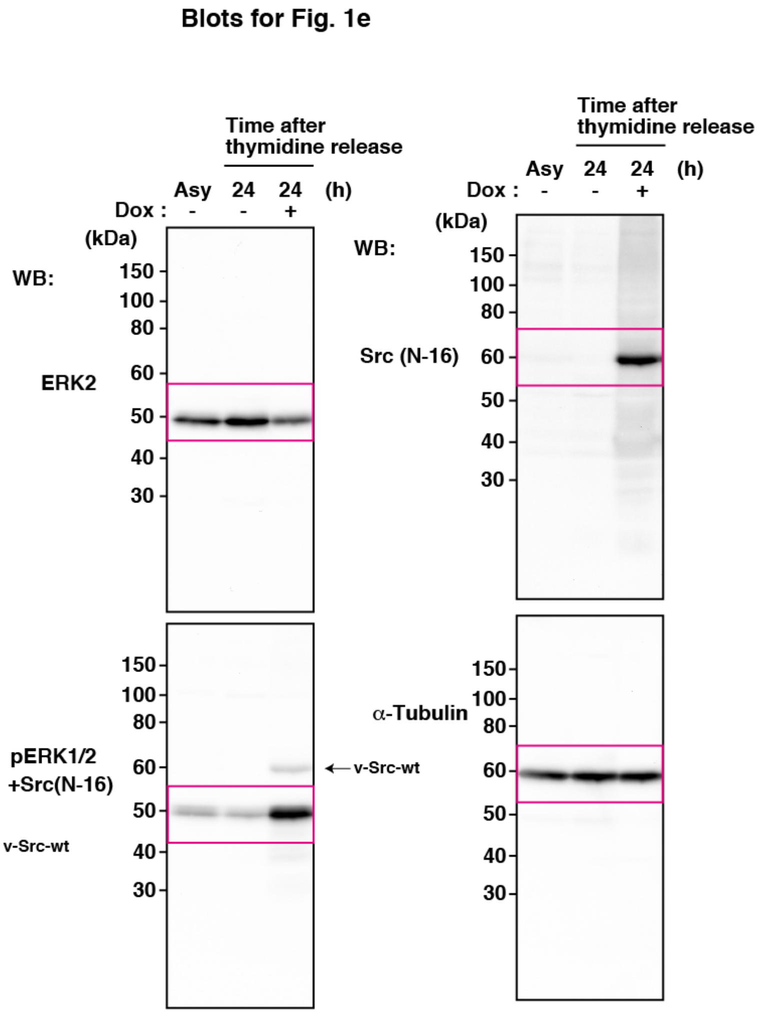

Supplementary Figure S3. Full-length blots. **(a)** Full-length blots for Fig. 1c. **(b)** Full-length blots for Fig. 1e.

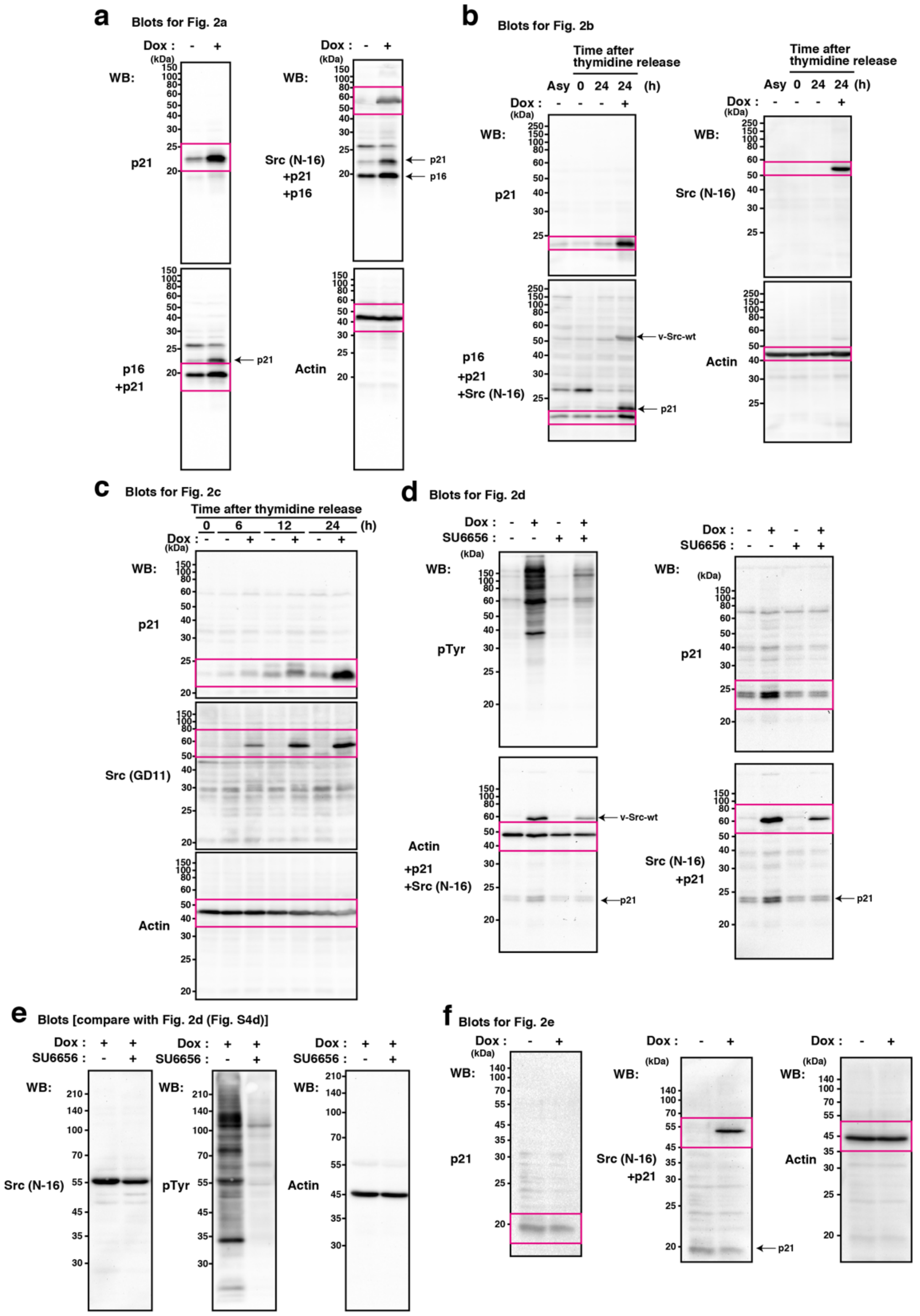

Supplementary Figure S4. Full-length blots.

(a) Full-length blots for Fig. 2a. **(b)** Full-length blots for Fig. 2b. **(c)** Full-length blots for Fig. 2c. **(d)** Full-length blots for Fig. 2d. **(e)** HeLa S3/TR/v-Src-wt cells were cultured for 6 h with 1 µg/ml Dox in the presence or absence of 10 µM SU6656. Whole cell lysates were analyzed by Western blotting (WB) using anti-Src (N-16), anti-pTyr, and anti-actin (loading control) antibodies. Unlike Fig. 2d (Fig. S4d), SU6656 treatment did not show an apparent reduction in the level of v-Src expression, possibly due to variations of each sample for some reason. **(f)** Full-length blots for Fig. 2e.

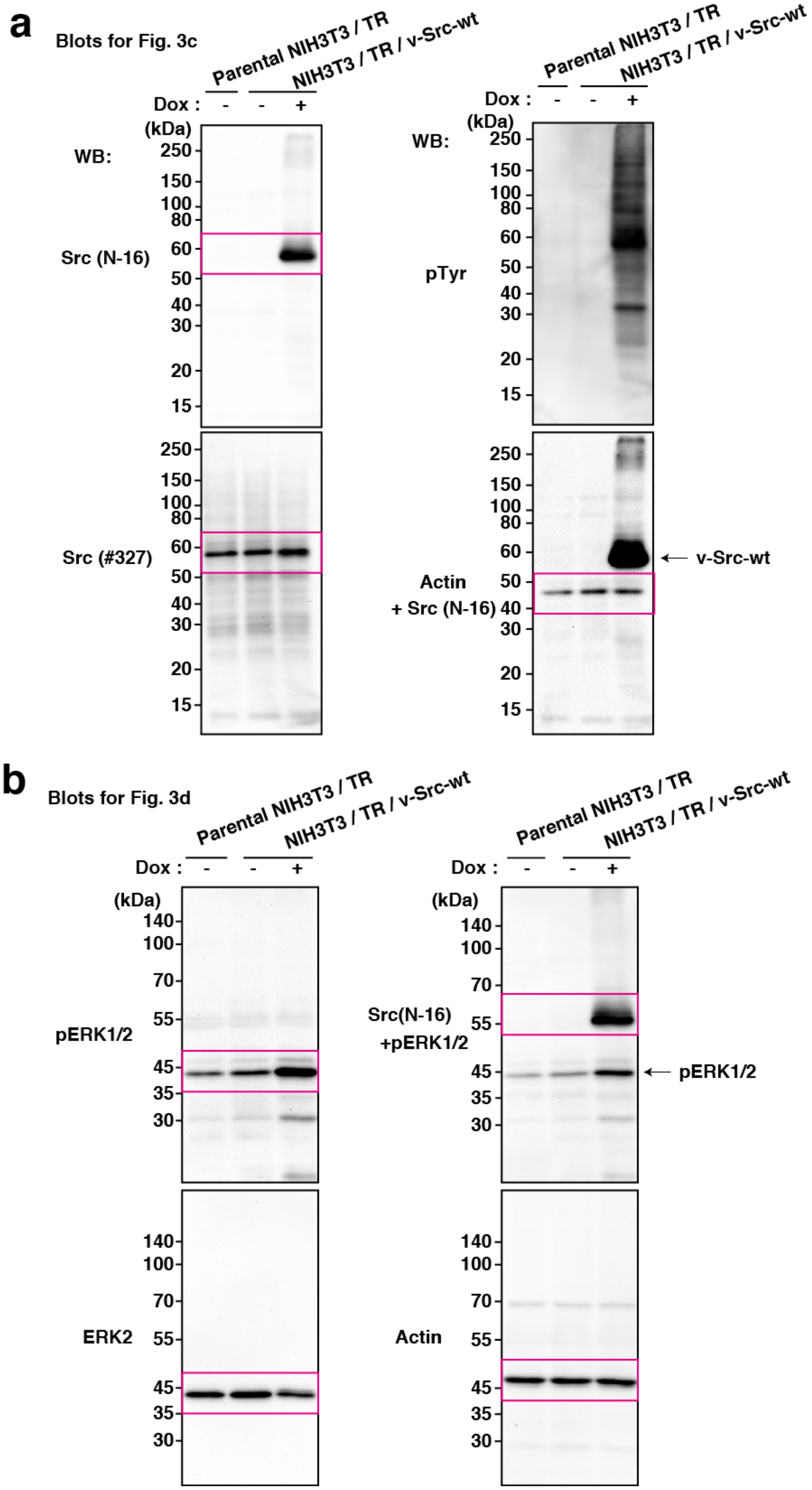

Supplementary Figure S5. Full-length blots.

(a) Full-length blots for Fig. 3c. **(b)** Full-length blots for Fig. 3d.

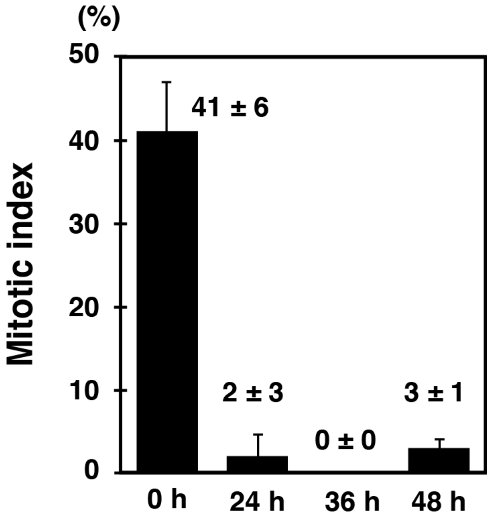

Supplementary Figure S6. Mitotic index of v-Src-expressing cells.
NIH3T3/v-Src-wt cells were treated with 1 µg/ml Dox for the indicated times and 100 ng/ml nocodazole for the last 12 h. Cells were fixed and stained with propidium iodide. Mitotic cells were counted (mitotic index). Results represent means ± S.D. from 4 high-power fields.

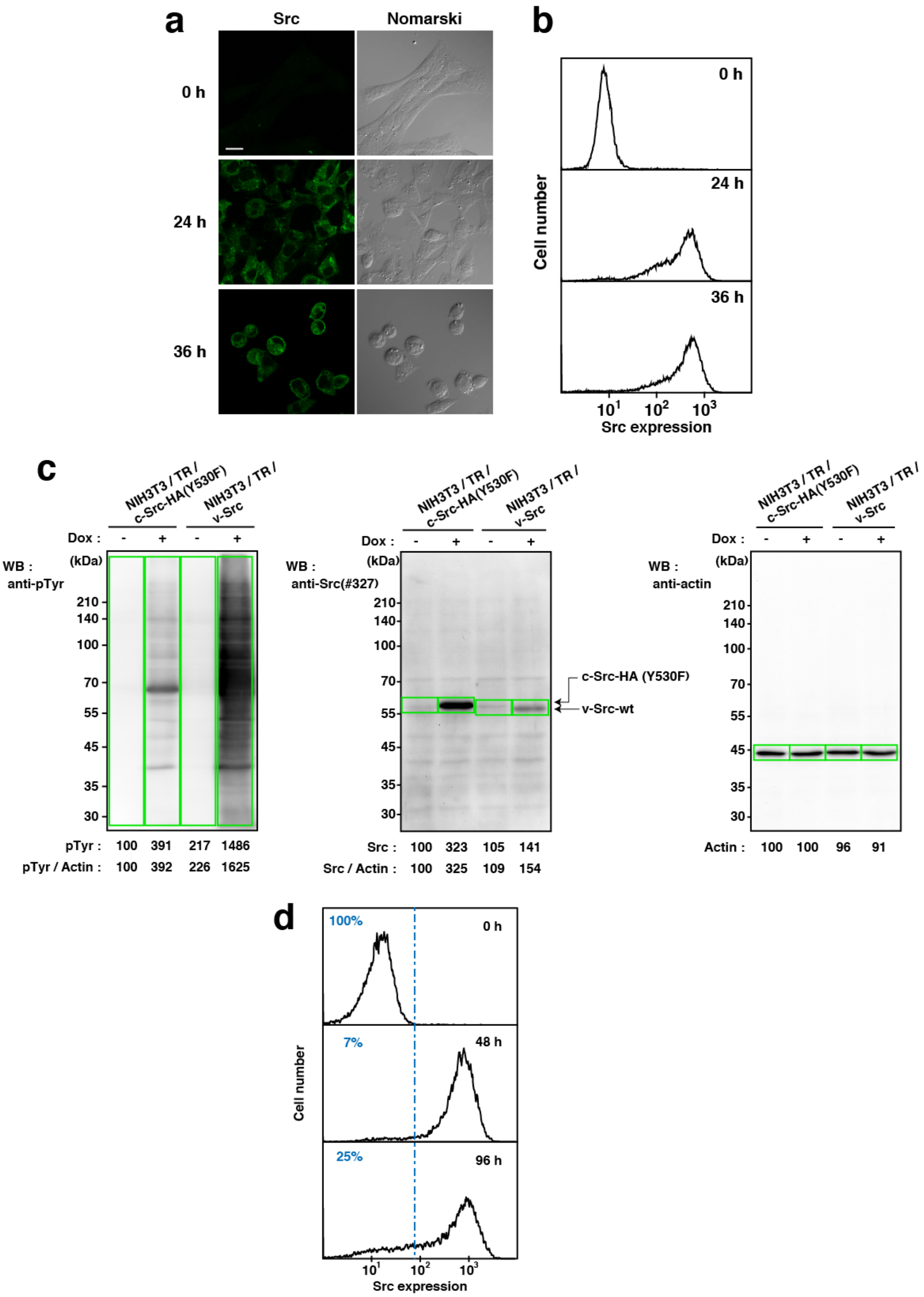

Supplementary Figure S7. The difference in the kinase activity between v-Src and activated c-Src.

(a, b) NIH3T3/TR/c-Src-HA(Y530F) cells were cultured for 24 h or 36 h with 1 µg /ml Dox. Cells were stained with anti-Src (#327) antibody. **(a)** Confocal microscopic images and **(b)** flow cytometric histograms were shown. Scale bars, 20 µm. **(c)** NIH3T3/TR/c-Src-HA(Y530F) and NIH3T3/TR/v-Src cells were cultured for 24 h with or without 1 µg/ml Dox. Whole cell lysates were analyzed by Western blotting (WB) using anti-phosphotyrosine (pTyr), anti-Src (#327) and anti-actin antibodies. Full-length blots are presented. **(d)** NIH3T3/TR/c-Src-HA(Y530F) cells were cultured for 48 h or 96 h with 1 µg/ml Dox. Cells were stained with anti-Src (#327) antibody.

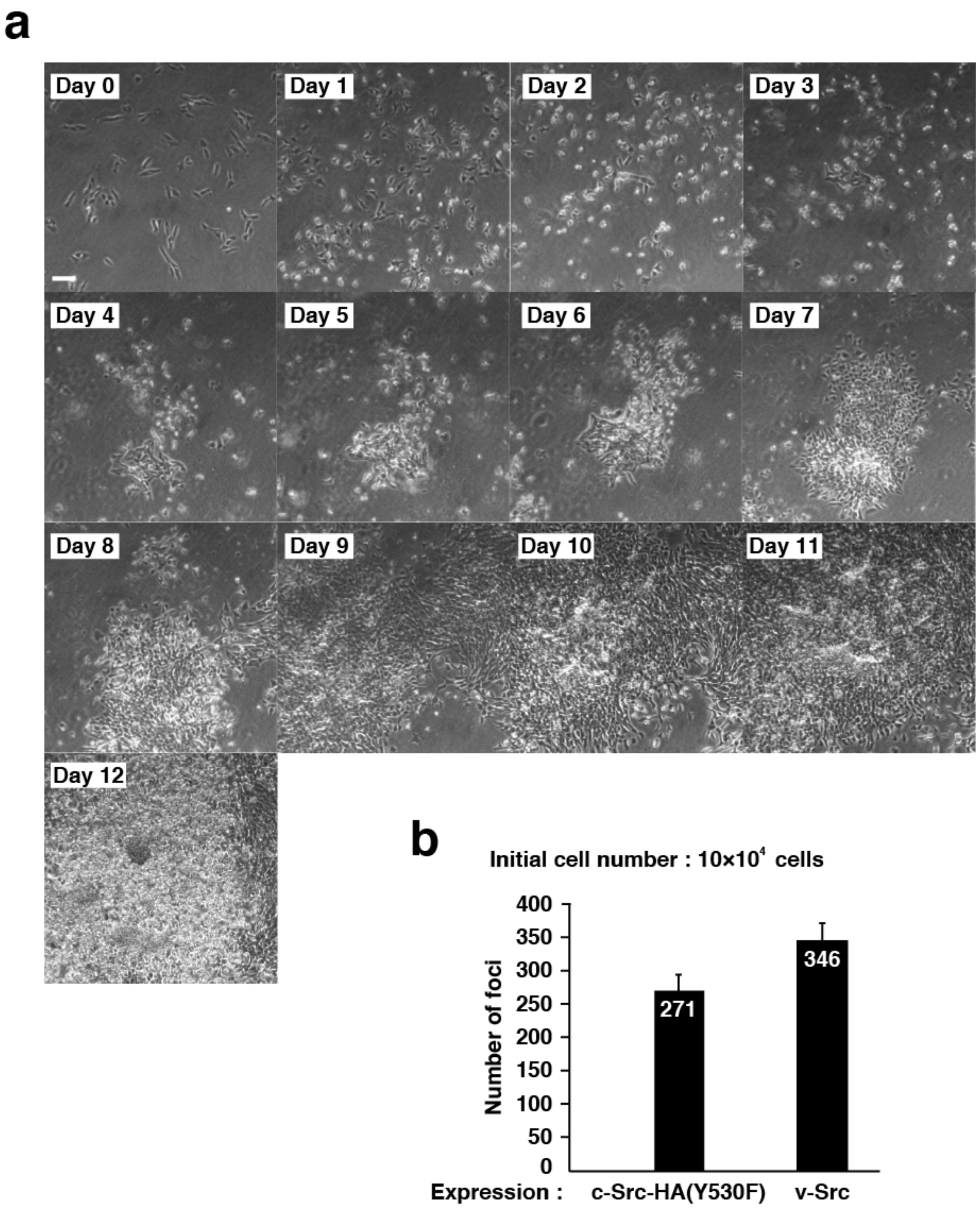

Supplementary Figure S8.   Induction of colony formation by activated c-Src.

(a) NIH3T3/TR/c-Src-HA(Y530F) cells (1 x 10^5 cells / 60-mm dish) were cultured for 1 day and treated with 1 µg/ml Dox for 12 days, and the same field images were continuously observed for 12 days by phase-contrast microscopy. Scale bar, 100 µm. **(b)** Colony focus formation assay. NIH3T3/TR/c-Src-HA(Y530F) and NIH3T3/TR/v-Src-wt cells were treated with 1 µg/ml Dox for 12 days. The number of piled-up transformed foci was counted. Results represent means ± S.D. from three independent experiments.
